# Empirically driven transdiagnostic stages in the development of mood, anxiety and psychotic symptoms in a cohort of youth followed from birth

**DOI:** 10.1038/s41398-023-02396-4

**Published:** 2023-03-29

**Authors:** Aswin Ratheesh, Dylan Hammond, Caroline Gao, Steven Marwaha, Andrew Thompson, Jessica Hartmann, Christopher Davey, Stanley Zammit, Michael Berk, Patrick McGorry, Barnaby Nelson

**Affiliations:** 1 Orygen, Parkville, Australia; 2 Centre for Youth Mental Health, University of Melbourne, Melbourne, Australia; 3 Institute for Mental Health, School of Psychology, University of Birmingham, Birmingham, UK; 4 Division of Mental Health and Wellbeing, Warwick Medical school, University of Warwick, Coventry, England; 5 Department of Psychiatry, University of Melbourne, Melbourne, Australia; 6 Population Health Sciences, Bristol Medical School, University of Bristol, Bristol, UK; 7 MRC Centre for Neuropsychiatric Genetics and Genomics, School of Medicine, Cardiff University, Cardiff, UK; 8 Institute for Mental and Physical Health and Clinical Translation (IMPACT), Deakin University, Barwon Health, Geelong, Australia

**Keywords:** Psychiatric disorders, Depression, Schizophrenia, Bipolar disorder

## Abstract

Staging models with transdiagnostic validity across mood, psychotic, and anxiety disorders could advance early intervention efforts as well as our understanding of the common underpinnings of such psychopathology. However, there are few well-supported operationalisations for such transdiagnostic models, particularly in community-based samples. We aimed to explore the inter-relationships among mood, psychotic, and anxiety symptom stages, and their common risk factors to develop data-informed transdiagnostic stages. We included participants from the Avon Longitudinal Study of Parents and Children (ALSPAC), a prospective ongoing birth cohort study. We developed operational thresholds for stages of depressive, hypomanic, anxiety, and psychotic symptoms based on the existing literature, refined further by expert consensus. We selected 1b level as the primary stage or outcome of interest. This represents moderate symptoms that are likely to be associated with the onset of the need for clinical mental health care. We used questionnaire and clinic data completed by young people ages 18 and 21 years. We used descriptive methods and network analyses to examine the overlap among Stage 1b psychopathology. We then examined the patterns of relationships between several risk factors and 1b stages using logistic regressions. Among 3269 young people with data available to determine all symptom stages, 64.3% were female and 96% Caucasian. Descriptive and network analyses indicated that 1b level depressive, anxiety, and psychotic symptom stages were inter-related while hypomania was not. Similarly, anxiety, depressive, and psychotic 1b stages were associated with the female sex, more emotional and behavioral difficulties in early adolescence, and life events in late adolescence. Hypomania was not related to any of these risk factors. Given their inter-relationships and similar risk factors, anxiety, psychotic and depressive, symptoms could be combined to form a transdiagnostic stage in this cohort. Such empirical transdiagnostic stages could help with prognostication and indicated prevention in youth mental health.

## Introduction

Concurrent and longitudinal comorbidity across mental disorders [1, 2] and their common risk factors have led to the development of transdiagnostic models for early intervention for serious mood and psychotic disorders [3]. Such transdiagnostic approaches are significant in the context of a new wave of intervention trials [4, 5], especially in primary care settings [6]. It has also been proposed that pooled transdiagnostic outcomes help address the challenges of statistical power in prevention trials [7], particularly if heterogeneity is minimised. In such prevention or early intervention efforts for severe mental disorders, ‘staging’ could provide a useful framework for identifying the need for early or preventive care [8]. While staging models have been conceptualised for several individual disorders [9], cross-cutting staging frameworks [10] that span mood and psychotic disorders as well as an international consensus statement on transdiagnostic staging in youth [11] have also been proposed. Such transdiagnostic staging models describe Stages 0 through 4, comprising asymptomatic familial risk (Stage 0), Stage 1a as the presence of mild or non-specific symptoms of mental disorders, 1b as the presence of moderate but subthreshold symptoms, Stage 2 as full-threshold disorder with moderate to severe symptoms, followed by stages of recurrence and refractoriness (Stages 3 and 4 respectively).

Within these stages, Stage 1 is critical from an indicated prevention perspective as it captures the period before illness is established and where targeted preventive interventions could be deployed. Trans-diagnostic approaches may also have greater validity in Stage 1 given the likelihood of greater overlap of syndromes [12], before the hypothesized differentiation of disorders into more typical trajectories in Stages 2 to 4. In such early stages, the interventions necessary are less clear and may be effective for several symptom domains. For example, young adults with significant depressive, anxiety, or attenuated psychotic symptoms (equivalent to Stage 1b) benefit from transdiagnostic interventions such as cognitive behavioural therapy [13]. While this intervention may remain valuable in stages 2 to 4, more specific treatments such as antipsychotic medications, mood stabilisers, and specific psychological therapies (e.g., interpersonal and social rhythms therapy in established bipolar disorder [14]) have firmer evidence and may have a better risk-benefit profile. Therefore, while transdiagnostic conceptualisations or interventions do not negate the value of disorder-specific interventions in established mental illness, the fluid and overlapping presentations of mental disorders in early illness course [12, 15] means that transdiagnostic approaches may be particularly helpful in early symptom stages. Within Stage 1, it is likely that Stage 1b represents the onset of the need for clinical mental health care. Clinicians reported that an overwhelming majority of those at Stage 1b should receive traditional mental health interventions such as face-to-face therapeutic consultations and family engagement [16]. Thus, Stage 1b could be considered an ‘onset stage’ with respect to the need for mental health care.

In such early onset stages, combining several mental disorders into a ‘transdiagnostic 1b stage’ could be useful for future research. Pooled transdiagnostic stages could help understand early pathophysiological processes common to mental disorders; help determine the onset of the need for care from a public mental health perspective; or as an outcome of interest in indicated prevention trials. However, there is little empirical data that can help define this stage. The studies empirically examining staging have operationalised the stages for individual disorders such as depression [17], bipolar disorder [18, 19], anxiety [20], and psychosis [21] in clinical samples. In addition to these, transdiagnostic staging models have been developed using clinical consensus approaches [22] or using operationalised criteria [23, 24] in clinical help-seeking samples. To date, there have been no studies examining the operationalisation of staging in community samples of youth. Such cohorts are important as symptom stages can be examined without selection biases present in clinical populations [25, 26] and improve generalisability of the staging construct to community samples. Finally, such community cohorts also allow us to examine the development of these stages prospectively without recall bias.

In community-based cohorts of youth, two key parameters that could help operationalise transdiagnostic stages across mood, anxiety, and psychotic disorders are: a) inter-relationships among the individual symptom stages and b) their common risk factors. In considering the inter-relationships among stages, two previously proposed transdiagnostic models [3, 11] suggested pooling severe mood and psychotic disorders. However, it is not clear if all mood symptoms (e.g., depression, hypomania, or mania) overlap similarly with psychotic symptoms to justify their being pooled into a transdiagnostic stage. In these models, the role of anxiety disorders is also unclear. Staging models for psychosis had considered anxiety symptoms to be ‘non-specific symptoms’, while it is clear that anxiety contributes to impairment and likely lead to Stage 1b or 2 syndromes in themselves [20].

Examining the inter-relationships among depressive, hypomanic, psychotic, and anxiety symptom stages is necessary to determine whether pooled transdiagnostic stages of mental health symptoms can be meaningfully defined. Similarly, common risk factors could also support the utility of pooled transdiagnostic stages as these risk factors could be targeted in prevention trials. Risk factors such as ethnicity [27, 28], social class [29], childhood adversity [30], neurocognition [31], life events [32], and substance use [33] have been associated with onset, severity or persistence of major mental disorders such as psychosis, depression, bipolar disorder, and anxiety. Similarly, a family history of mental disorders increases the risk of the same mental disorder, as well as other disorders among probands [34]. Finally, early life emotional and behavioral difficulties are associated with poorer mental health in adulthood [35]. If these risk factors are associated with the onset of Stage 1b level symptoms of some disorders, but not others, this may also help understand how stages may be pooled. We, therefore, aimed to explore the patterns of common risk factors as well as the inter-relationships among operationally defined 1b stages for depressive, hypomanic, psychotic, and anxiety symptoms in a community sample of youth followed from birth. A priori, we decided not to test specific hypotheses given the relatively nascent empirical research in the field of transdiagnostic staging.

## Methods

Ethical approval for the study was obtained from the ALSPAC Ethics and Law Committee and the Local Research Ethics Committee. Informed consent for the use of data was obtained from participants following the recommendations of the ALSPAC Ethics and Law Committee at the time.

### Population

We selected the Avon Longitudinal Study of Parents and Children (ALSPAC) which is an ongoing prospective cohort study that enrolled pregnant women residing in Avon, UK, with expected delivery dates between April 1991 and December 1992. These women and their children have been followed ever since. The cohort profile and the study methods have been described previously [36, 37], including additional participants from later phases of recruitment [38]. The initial number of pregnancies enrolled is 14,541, and of these initial pregnancies, there were 13,988 children who were alive at 1 year of age. When the oldest children were approximately 7 years of age, an attempt was made to bolster the initial sample with eligible cases who had failed to join the study originally. Participants who were originally eligible, but not included were also allowed to start participating later in adolescence or young adulthood. This led to a final sample size of 15,645. Please note that the study website contains details of all the data that is available through a fully searchable data dictionary and variable search tool (http://www.bristol.ac.uk/alspac/researchers/our-data/). Due to the attrition of participants from birth to young adulthood, complete data were available only for subsets of participants who participated in various follow-up methods described below. In the current study, we included participants for whom sufficient data were available on the required measures to determine Stage 1b for all symptom types. Please see Supplementary Figure 2 for details of participants included and data available.

### Measures

**Fig. 1:**
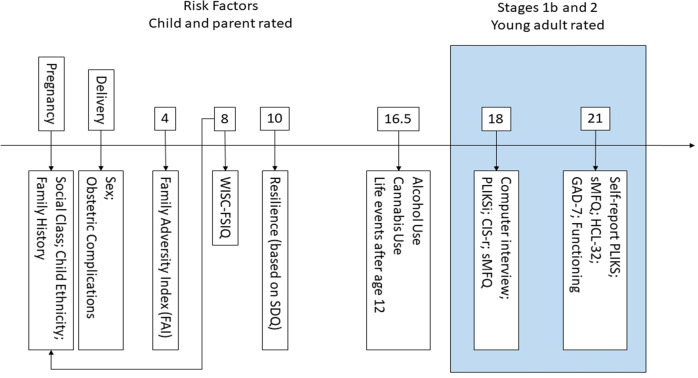
Timeline of measures included in the current study. WISC-FSIQ Weschler Intelligence Scale for Children, Full-Scale Intelligence Quotient, SDQ Strengths and Difficulties Questionnaire, sMFQ short Moods and Feelings Questionnaire, PLIKS Psychosis Like Symptoms, Interview or Questionnaire, CIS-r Clinical Interview Schedule- revised, HCL-32 Hypomania Checklist 32.

We examined variables at several time points from birth to young adulthood (Fig. 1). The participants’ physical and mental health data in early adolescence and young adulthood were obtained using two methods: a) self-report questionnaires completed by either the young person themselves or their parents and b) ‘focus clinics’ which included interview-based and self-report assessments conducted while attending such a clinic. In addition, hospital clinical data were linked to complete the obstetric and perinatal data.

#### Staging variables

The measures used to determine stage 1b disorders comprised: the short Moods and Feelings Questionnaire (s-MFQ [39]), the Generalized Anxiety Disorder-7 (GAD-7 [40]), Psychosis Like Symptoms (PLIKS determined by self-report and computer-based interview [41]), the Hypomania Checklist (HCL-32 [42]) as well as additional self-report items assessing the impact of HCL-32 symptoms on functioning, the revised Clinical Interview Schedule (CIS-r [43]), and items assessing the functional impact of emotional disorder from the Medical Outcomes Study Short Form (SF-36 [44]).

### Operational definitions of Stages 1b and 2

We operationalised clinical stages (Table 1) based on the model proposed by Scott and colleagues [3], further refined by consensus amongst experienced psychiatrists and psychologists involved in this project (AR, AT, SM, CD, BN, and PM). This approach allowed us to examine meaningful clinical outcomes, informed by the principles of transdiagnostic staging [11] and using anchor points from DSM and ICD diagnostic criteria when possible.

**Table 1:** Summary of criteria used to determine Stage 1b and Stage 2+.

Disorder	Stage 1b	Stage 2+
Depression	*Recurrent/persistent depression present at BOTH time-points:* - Age 18: Moderate MDE on CIS-r. - Age 21: s-MFQ score ≥11 and impairment in functioning at least ‘some of the time’ in the previous month. OR *Severe MDE at EITHER time point:* - Age 18: Severe MDE on CIS-r. - Age 21: sMFQ score ≥20 and impairment in functioning at least ‘some of the time’ in the previous month.	*Severe and impairing depression at either time-point:* - Age 18: Severe MDE on CIS-r, and decline in functioning compared to previous year (‘worse’ or “much worse’) - Age 20: s-MFQ score ≥20 and impairment in functioning ‘all of the time’ in the previous month.
Bipolar	*Lifetime hypomanic episode:* - Age 21: HCL-32 ≥14 score, with highs lasting for at least 4 days, changes observed by friends or family as either positive or negative, and at least some negative functional impact - Age 18: (insufficient data available).	*Bipolar I* *Lifetime manic episode* - Age 21: HCL-32 ≥ 14 score, with highs lasting for at least 7 days, changes observed by friends or family as negative, and some negative functional. OR *Bipolar II* *Lifetime hypomanic episode (See Stage 1b Bipolar)* AND *Moderate to severe depressive episode at either time point:* - Age 18: Moderate or severe depressive episode on CIS-r. - Age 21: s-MFQ ≥ 11, with recent functional impairment most of the time in last month.
Psychosis	*Psychotic symptoms at either time-point:* - Age 18 (trained rater + self-report): One or more definite primary* psychotic symptom present at least monthly in the last 6 months and causing distress, help-seeking, or functional impairment. - Age 21 (self-report only): One or more definite primary* psychotic symptom, in the last 6 months, at least monthly, associated with impairment in functioning.	*Threshold psychosis with impairment:* - Age 18 (trained rater + self-report): One or more definite primary* psychotic symptom present most days in the last 6 months and causing considerable distress with ‘much worse’ function relative to the previous year.
Anxiety	*Recurrent/persistent anxiety present at BOTH time-points:* - Age 18: Generalised anxiety or phobia CIS-r ≥ 2. - Age 21: Significant anxiety symptoms, GAD-7 ≥ 10. OR *Severe/impairing anxiety at EITHER time point:* - Age 18: Generalised anxiety or phobia CIS-r ≥ 3. - Age 21: Significant anxiety symptoms, GAD-7 ≥ 10, AND recent functional impairment due to emotional disorder (all or most of the time). Higher threshold anxiety *Recurrent/persistent anxiety present at BOTH time-points as defined above* OR *Higher threshold severe/impairing anxiety at EITHER time point:* - Age 18: Generalised anxiety or phobia CIS-r ≥ 4. - Age 21: Significant anxiety symptoms, GAD-7 ≥ 15, AND recent functional impairment due to emotional disorder (most of the time).	*Severe anxiety with functional impairment at EITHER time-point:* - Age 18: generalised anxiety or phobia CIS-r ≥ 4 and impairment in functioning relative to the previous year (‘worse’ or ‘much worse’). - Age 21: Significant anxiety symptoms, GAD-7 ≥ 15, and recent functional impairment due to emotional disorder (all of the time).

*sMFQ* short Moods and Feelings Questionnaire, *CIS-r* Clinical Interview Schedule- revised, *HCL-32* Hypomania Checklist 32, *MDE* major Depression Episode; * Primary refers to symptoms that were not reported to be related to sleep, fever, or drug use.

The outcomes were the presence of Stage 1b level of mental health symptoms in young adulthood (ages 18–21 years), relating to depression, anxiety, psychosis, and hypomania. This age period corresponded to the peak age of onset of a range of mental disorders [45]. To meet criteria for Stage 1b, we specified that significant symptoms of psychosis, hypomania, or moderate to severe depression or anxiety should be present, along with indicators of significant impact of these symptoms. The impact was determined based on functional impairment, help-seeking, substantial distress, or self-reported impact, in line with the proposed staging model [3]. In order to translate staging models developed in clinical populations to a community cohort such as ALSPAC, two key modifications were necessary compared with published guidelines on transdiagnostic staging outlined below. First, across all disorder definitions, a more stringent threshold was adopted to ensure that measures used for screening could be utilised for case finding and thus decrease the risk of false positives. This ensured that all Stage 1b definitions were of likely clinical consequence, although at a higher threshold than what has previously been recommended in staging models. Second, we included the requirement for persistence or recurrence for ‘common mental disorders’ such as depression and anxiety in order to achieve a similar prevalence as low prevalence mental health conditions such as hypomania and psychosis. This translates the differences in help-seeking or referral for anxiety and depression (compared to that for psychosis or bipolar disorder) in clinical settings where staging is commonly applied. Additionally, this was necessary to explore associations and common risk factors across common and low-prevalence mental health symptom stages with relatively similar power. The rationale is detailed in Supplementary Material.

Further, we defined Stage 2 or more (Stage 2+) level symptoms during this period so that we could identify individuals with a more advanced stage of symptoms and exclude them from analysis pertaining to Stage 1b. Primary definitions of Stage 1b and Stage 2+ symptoms are outlined below and in Table 1, with further details on operational definitions available in the Supplementary Material.

#### Stage 1b psychosis

1b psychosis was defined as the presence of definite psychotic symptoms in the last 6 months that were not related to sleep, fever, or drug use, at least monthly, and associated with distress, impairment in functioning, or help-seeking.

#### Stage 1b hypomania

1b hypomania was defined as the presence of significant hypomanic symptoms based on their HCL-32 score ≥14 score [46] occurring within the same period, lasting 4 days or more, observed by friends or family, and associated with a negative functional impact.

#### Stage 1b depression

1b depression included the presence of moderate major depression at both time points (indicating recurrent or persistent depression) or the presence of severe major depression at either time point. Moderate major depression was identified based on a syndromal definition on the CIS-r (age 18 years), or an sMFQ score ≥11 at 21 years with concurrent impairment in functioning based on three items on SF-36. Severe major depression was similarly identified through CIS-r criteria at age 18 years or an sMFQ score ≥20 [47] at age 21 with severe concurrent impairment in functioning on the same SF-36 items.

#### Stage 1b anxiety

Similar to 1b depression, 1b anxiety included the presence of recurrent or persistent moderate anxiety (i.e., moderate anxiety present at both time points) or the presence of severe and impairing anxiety at either time point. Moderate anxiety was identified at age 18 based on CIS-R ratings of 2 or more on overall anxiety or phobia, while at age 21 moderate anxiety was defined as a score of 10 or more on the GAD-7 [40]. Similarly, severe and impairing anxiety was identified at age 18 based on CIS-r ratings (3 or higher) and at age 21 with a combination of symptom severity on GAD-7 (≥10) combined with concurrent impairment in functioning all or most of the time on the same SF-36 items.

Given the expected higher prevalence of 1b anxiety compared to other disorders, we explored a ‘higher threshold Stage 1b anxiety’ definition which was likely to be similar in prevalence to that of depression, psychosis, and hypomania. In this definition, recurrent or persistent anxiety was defined in the same manner, but severe and impairing anxiety was determined based on a higher symptom threshold. At age 18, we used a higher CIS-r rating of ≥4 and at age 21 we utilized a higher threshold on GAD-7 (≥15) combined with concurrent impairment in functioning all or most of the time, again on the same SF-36 items.

##### Stage 2 (exclusion criteria)

For each symptom type, we also developed definitions of Stages 2 or more (Stage 2+) definitions included severe and impairing psychotic symptoms, symptoms meeting criteria for mania as well as severe and impairing depression or anxiety. The operational definitions are summarised in Table 1 and further detailed in Supplementary methods.

##### Risk factors

We explored a broad range of risk factors determined between 0 and 16 years of age, selected for their known association with mental disorders and availability in ALSPAC. These included sex at birth, ethnicity (Caucasian or non-Caucasian), social class (parental occupation reported during pregnancy), obstetric complications (resuscitation at birth), family history of mental disorders (severe depression or schizophrenia in first-degree relatives), cognitive ability as measured by the Full-Scale Intelligence Quotient from the Weschler Intelligence Scale for Children (WISC-FSIQ [48]), early life psycho-social adversity (score of one or more on the short-form of the Family Adversity Index, FAI [49]), hazardous alcohol use (score of 8 or more on the Alcohol Use Disorders Identification Test, AUDIT [50]), any cannabis use (measured with Cannabis Use Screening Test [51]), life events self-reported as ‘highly unpleasant’ (parental relationships, peer relationships, difficulties at school, and losses). Additionally, we also used data from the Strengths and Difficulties Questionnaire [52] completed by parents at age 10, as a measure of parent-rated symptoms across emotional and behavioral domains. Details on operationalisation of risk factors and the rationale for their inclusion are also provided in Supplementary Methods.

### Analysis

To investigate relationships among Stage 1b disorders, and describe the prevalence of Stages 1b and 2, we used a mixture of visual and analytic methods within the R programming language (version 3.6.3) [53]. Euler diagrams were used to investigate the relative contribution of each disorder to a combined stage definition. Euler diagrams are similar to Venn diagrams, though with the area of sections proportional to the underlying count data. We used the *eulerr* package (version 6.1.0 [54]) to estimate and plot diagrams.

To investigate relationships among disorders within Stage 1b, we used a network graphic approach, modelling interactions between disorders using the regression-based Ising model, as described in Van Borkulo, Borsboom [55] and implemented in the *bootnet* package (version 1.5 [56]). Briefly, in this model, each node (i.e., each Stage 1b disorder) is regressed on all other nodes in turn and resultant edge-weights are the average of the two regression coefficients (e.g., A on B and B on A). Unimportant edges are shrunk to zero using regularisation. Further details are provided in Supplementary Methods. To assess the accuracy and stability of edges within the network, we conducted non-parametric boot-strap analyses, both with and without case-dropping. Although limited by the number of indicator variables, we also explored the presence of at least one latent dimension across the four categorical symptom stages in factor analysis using a Weighted Least Squares Mean and Variance Adjusted (WLSMV) estimator (Mplus V8.0).

Finally, to explore the pattern of risk factors predictive of each Stage 1b disorder, we regressed each disorder onto each risk factor using simple logistic regressions, extracting and comparing odds-ratios and 95% confidence intervals. Multiple regressions or temporally ordered analyses accounting for confounding or mediation were not possible due to the small cell sizes for individual symptom stages, particularly psychosis and hypomania.

#### Missing data

For all analyses, we used participants with enough data to determine all four Stage 1b disorders. Where participants were missing data on prior risk variables, we used multiple imputation by chained equations (MICE), as implemented in the *mice* package [57]. We imputed 70 complete datasets which we used for the logistic regression analyses of Stage 1b disorders onto prior risks, averaging across parameter estimates according to Rubin’s rules to arrive at plausible final estimates. We additionally compared the results obtained when imputing data to those obtained when using pairwise deletion of cases.

## Results

From a total eligible sample of 15,645 in the ALSPAC cohort, 3346 (21.4%) had enough data to determine all Stage 1b disorders, while 2326 (14.9%) had partial Stage 1b data and 9973 (63.7%) had no Stage 1b data. Those with only partial or no data on Stage 1b disorders were more likely to be male, from a lower social class by parental occupation in-utero, have a lower full-scale IQ in childhood, and more likely to engage in harmful drinking in adolescence. For a more complete breakdown of demographic, risk, and clinical variables by missingness, please see Supplementary Table 2 and Supplementary Fig 2. Of those with complete Stage 1b data, 77 (2.3%) individuals also met criteria for Stage 2 disorder. After excluding these individuals, a total sample of 3269 remained for the following complete-case analysis.

The included sample (*N* = 3269) were predominantly female (64.3%), white (96%), and from families with high socioeconomic background by parental occupation (59.5%). A high proportion reported knowing a first-degree relative with a history of severe depression (19.3%), while only very few reported a first-degree relative with schizophrenia (0.34%). By age 16, roughly a quarter (25.5%) of the sample had tried cannabis, 34.9% scored above thresholds for screening harmful drinking on the AUDIT, and the majority (60.6%) reported having experienced a highly unpleasant life event.

### Prevalence of individual disorders and their overlap

In our sample, 11.3% symptoms were at or above our threshold for Stage 1b disorder (8.7% when using higher thresholds for anxiety). The most prevalent of the disorders at Stage 1b was anxiety, ~3.3–5.6 times the other disorders (Table 2). However, using a higher threshold for Stage 1b anxiety predictably meant that its prevalence was less disproportionate to that of other Stage 1b disorders (1.9–3.3 times greater than other disorders).

**Table 2:** Prevalence of Stages 1b and 2+ in the included sample (*N* = 3343).

Disorder	Stage 1b *n* (%)	Stage 2+ *n* (%)
Depression	59 (1.76%)	37 (1.11%)
Hypomania/Bipolar	39 (1.17%)	15 (0.45%)
Psychosis	34 (1.02%)	<5 (<1%)
Anxiety	225 (6.72%)	42 (1.26%)
Anxiety higher threshold	111 (3.32%)	

**Fig. 2:**
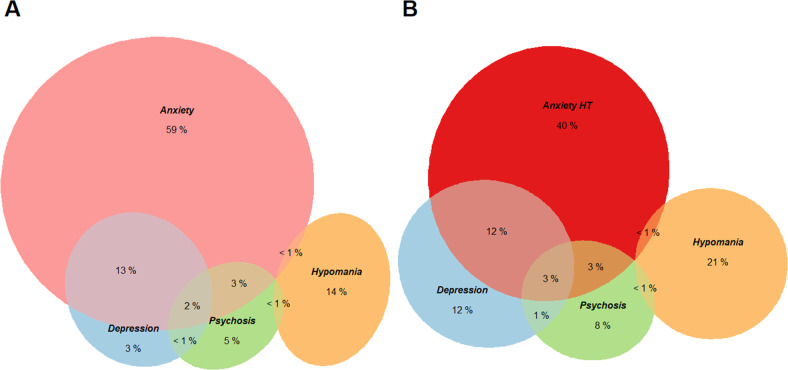
Euler diagrams representing the composition of Stage 1b symptoms. **A** Stage 1b using the lower threshold for anxiety and **B** a higher threshold for anxiety. Numeric labels represent the proportion each group or intersection contributes to the stage. Sample sizes are 264 and 179, respectively.

From the Euler diagrams (Fig. 2), anxiety continued as a determining factor in threshold mental health issues; those with anxiety alone made up 59% of Stage 1b, though this proportion diminished (40%) when using a higher threshold for anxiety. Comorbidity was reasonably low with 19–20% of individuals with a Stage 1b disorder meeting criteria for a second disorder. Finally, while anxiety and depression had moderate to high rates of overlap with each other, they did not overlap as consistently with either psychosis or hypomania. Indeed, hypomania was clearly isolated from other disorders, with 95% of those with Stage 1b hypomania only meeting criteria for this stage 1b disorder.

### Inter-relationships among disorder-specific stages

**Fig. 3:**
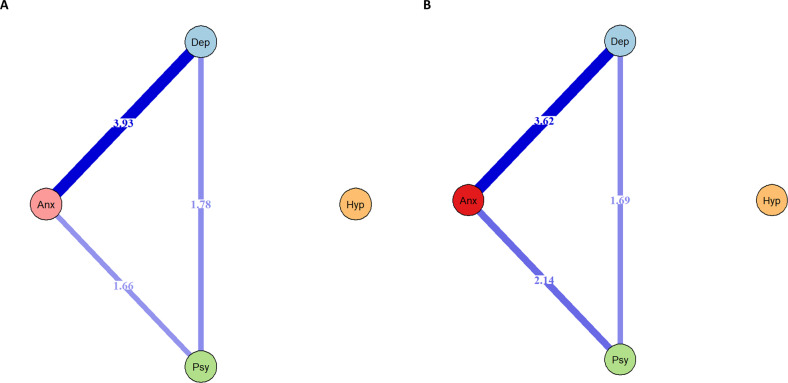
Inter-relationships between stage 1b symptoms determined using Ising networks. Gamma = 0.00; Rule = OR. Edge-weights are average beta coefficients. **A** Using lower-threshold Stage 1b anxiety; **B** using higher-threshold Stage1b anxiety. Hypomania not represented as unconnected to other Stage 1b symptom domains.

The regression-based Ising models (Fig. 3) were consistent with results from the Euler diagrams. While depression, anxiety, and psychosis were connected, hypomania was not related to any of the other indicators. The same was true when using either threshold for Stage 1b anxiety. When using a higher threshold for anxiety, the edge connecting anxiety to psychosis was more pronounced. In both networks, the correlation stability (CS) coefficients for edge-weights were reasonably robust, *CS* = 0.75 (see Supplementary Material). This indicates that these estimates are stable to changes in sample composition as similar edge weights were identified on dropping more than half of the cases from boot-strapped samples. Boot-strapped difference test found that while the edge between anxiety and depression was significantly stronger than others in the network, edges from psychosis to anxiety and depression were similar to each other.

The factor analytic model with a single latent dimension had good fit indices (chi-square 1.99, *df* = 2, *p* = 0.368; Tucker Lewis Index and Comparative Fit Index = 1; Root Mean Square of Approximation <0.05). The factor loadings for depression (fixed to 1), anxiety (0.87, SE = 0.15), and psychosis (0.64, SE = 0.1) were large and statistically significant (*p* < 0.001) while the factor loading for hypomania was not (−0.04, SE = 0.1, *p* = 0.682). Additional factors could not be identified due to the relatively few indicators available. Full results are presented in Supplementary Material.

### Risk factors for specific stage 1b disorders

**Fig. 4:**
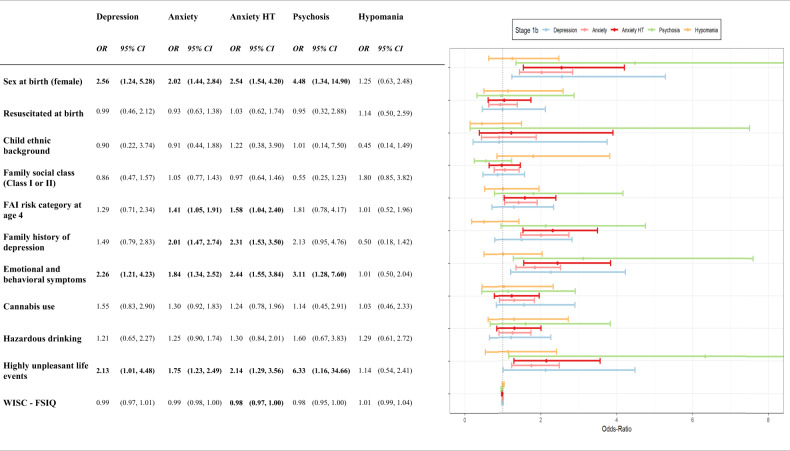
Associations between stage 1b stages and risk factors measured from birth. Odds-ratios and associated 95% confidence intervals from simple logistic regressions of disorder specific Stage 1b categories onto prior risk factors. Missing risk data has been imputed using Multiple Imputations using Chained Equations (MICE). HT Higher Threshold, FAI Family Adversity Index, WISC Weschler Intelligence Scale for Children, FSIQ Full-Scale Intelligence Quotient.

There were variable levels of missing data for the risk factors ranging from 0.1% for sex at birth to 44% for resuscitation status. Results from regression analyses using imputed data are presented in Fig. 4. Depression, anxiety, and psychosis had similar patterns of risk factors, while the same was not true of hypomania. Female sex at birth, more emotional and behavioral difficulties at age 10, and having experienced a highly unpleasant life event in mid-adolescence were associated with depressive, anxiety, and psychotic 1b Stages but not 1b hypomania. Stage 1b anxiety was also associated with a family history of depression. While we explored family history of schizophrenia as a risk factor, cell numbers were too low to estimate regression parameters. These results were similar to those using the same sample without imputation, as well as when conducting the same analyses in those with partial Stage 1b symptom data available (*N* = 3276–5545, Supplementary Tables 3 and 4).

## Discussion

In this study, we used stage definitions based on symptom severity and functioning to explore the interrelationships among 1b stage of anxiety, depression, psychosis, and hypomania and to explore patterns of risk factors common or unique to specific symptom stages. We identified that anxiety, depression, and psychosis 1b stages in young adulthood had independent relationships with each other, and a common latent dimension, while hypomania did not. Similarly, anxiety, depression, and psychosis had a pattern of common risk factors such as female sex, greater emotional and behavioral difficulties in early adolescence, as well as life events in mid-adolescence. There was little evidence that these risk factors were shared with hypomania.

The overlap in symptoms and inter-relationships between anxiety, depressive and psychotic symptoms [58–61] is well documented in community samples of adolescents and young adults. The presence of a significant overlap despite the use of instruments that measure the unique nature of these symptoms suggests that there is likely to be a transdiagnostic dimension underlying these disorders, especially in early stages of illness. Prior work from the ALSPAC cohort using data-driven modelling has also indicted that symptoms are distributed based on transdiagnostic distress rather than along unique diagnostic categories [62]. Our factor analytic approach also confirmed the presence of at least one latent dimension underlying depression, anxiety, and psychosis 1b stages, although the presence of other factors could not be clarified. Similarly, data from the Dunedin birth cohort strongly indicated the presence of a transdiagnostic vulnerability dimension (p-factor) using factor analytic methods [2]. Our finding that the edge-weights between depression and anxiety were stronger than those between psychosis and these disorders may also be due to the strong diagnostic overlap between depression and generalized anxiety. This also complements data from the Dunedin birth cohort study [2] where an internalising factor comprising depression and anxiety existed within the overall dimension of the higher-order level of the p-factor. Shared influence of both general psychopathology, as well as those unique for mood, anxiety, and psychotic disorder domains, has also been demonstrated using polygenic risk scores in the ALSPAC cohort [63]. These support the possibility of hierarchically ordered dimensions underlying psychopathology [64], which could be incorporated into or complement the staging approach.

The observation that hypomanic 1b stage was not related to other disorders is concordant with the clinical observation that hypomania is often not associated with distress [65], unlike anxiety, depression, or psychosis. Given that our network analysis utilised tuning parameters chosen to maximize the possibility of identifying non-zero edge weights, the absence of reciprocal relationships between hypomania and other disorders is significant. Factor analysis also indicated that hypomania 1b stage did not load on to a common latent dimension. However, the lack of relationships may also be due to measurement differences in relation to hypomanic symptoms. In ALSPAC, the 32-item Hypomania Checklist (HCL-32) measures lifetime hypomanic symptoms while anxiety, depression, and psychosis symptoms are measured cross-sectionally over weeks or months. Measurement differences between symptoms may also account for the limited overlap between symptoms in this study, compared with other transdiagnostic studies [2] which utilised the same measurement approach (e.g., semi-structured clinical interview) across different symptom domains. A unified approach to measuring symptoms (e.g., ordinal scales on structured diagnostic interviews) as well as cross-cutting measures of severity, distress, functioning and cognition may help develop better defined stages. This could be implemented in the future waves of ALSPAC, or other prospective cohort studies.

Several of the risk factors we identified to be associated with 1b stages of anxiety, depression, and psychosis have been previously identified at a symptom level in previous research using the ALSPAC [66] cohort and at a disorder level in previous observational studies [67]. Risk factors such as adverse life events have also been associated with psychosis [68], anxiety [69] and depression [70] in previous studies. While life events have been previously linked to episodes [71] or admissions [72] in those with established bipolar disorder, it is possible that their effects are less prominent or persistent on hypomanic episodes prior to illness establishment. It is also possible that hypomania may have unique risk factors, some of which may not have been examined in this study. It should also be noted that while the patterns of risk factors may be similar, previous work from this cohort indicates that there may be unique as well as shared risk associated with these symptom domains [66].

In our study, the observation of the higher incidence of anxiety compared with other symptom stages was expected based on community-based prevalence in adolescents and young adults [73]. The higher incidence could also explain the greater power to detect associations with some risk factors (e.g., family history of depression, family adversity) which could not be confirmed for other disorders. Anxiety symptoms also accounted for the majority of overlap between symptom stages. Whether anxiety vulnerability (or vulnerabilities) represents a more central construct within the transdiagnostic staging concept needs further evaluation. It is notable that the higher prevalence persisted despite our consensus-based approach where we set a higher threshold for common mental disorders such as anxiety and depression compared with low prevalence disorders such as psychosis or bipolar disorder. Such adaptations to the transdiagnostic models originally proposed for clinical samples are likely to be necessary to translate such models to community settings as we outline below.

Based on shared variance, inter-relationships, and common risk factors, we propose that several transdiagnostic onset stages can be proposed, in ALSPAC and other cohorts using similar measurement approaches.

(i) Common-construct Stage 1b: We suggest that 1b stages of depression, anxiety, and psychosis can be combined to represent a group that may have a shared underlying construct. Here, we use the term ‘common construct’ to indicate the possibility of latent structures or complex clusters [74] underlying the supra-ordinal construct of the transdiagnostic stage. Latent factors or clusters may be derived from such pooled stages which may help understand these constructs further. Such pooled stages may be a useful outcome in studies modelling the common risk pathways to onset as well as the shared neurobiology of these disorders.
(ii) Common-construct and similar-incidence Stage 1b: The incidence of anxiety stage 1b in our sample was higher relative to that of depression and psychosis, as expected. While psychotic symptoms are often prevalent in adolescents [75] or young adulthood, disorder level psychosis symptoms are much less prevalent by this age [76]. This could mean that in a common construct Stage 1b definition, risk factors (of relatively equal effect) that preferentially influence changes in anxiety could have a greater effect on the pooled outcome, compared to the risk factors common to all symptom stages or unique to other symptom stages. If it is necessary to limit this, we propose the use of the higher threshold anxiety Stage 1b in combination with 1b depression and psychoses. However, the use of such higher thresholds may be imposing artificial constraints on the prevalence of various mental health symptoms in the general population.
(iii) Utilitarian Stage 1b+: If the premise underlying the transdiagnostic stages is utility [11, 77] (e.g., prediction of any mental health symptom associated with need for care), it may also be reasonable to include all disorder stages in one transdiagnostic stage. Such a definition had a prevalence of 11% in the ALSPAC sample by age 21, indicating the potential to overcome some of the challenges of statistical power in risk prediction. Stages 2 or more could also be included in this stage definition if an upper threshold of severity or impairment is not necessary.

It may also be prudent to consider the limitations of such transdiagnostic stages in general, and in this cohort in particular. In coding combinations of symptom stages within a transdiagnostic model, it is important to note that pooled outcomes may be multi-dimensional including several symptoms or their clusters, functional impacts, or distress. Thus, a pooled transdiagnostic outcome may be akin to constructs such as health-related quality of life (including several domains of pain, mood, anxiety, and functioning). While offering substantial utility, such a construct could have some of the disadvantages of multidimensional constructs, particularly heterogeneity. If unidimensional outcomes are necessary, latent variable models should be considered within Common construct Stages outlined above. In the future, such heterogeneity may also be decreased by selecting subgroups of participants within the broader transdiagnostic stage, based on genetic, imaging, or other markers. It is possible that data-driven groups using a broad range of such markers within transdiagnostic stages may diminish the problems associated with validity in current psychiatric classification systems. Further, we limited our stage definitions to ages 18 and 21 years to enable us to include all symptom domains within the mood, anxiety, and psychotic disorders. However, our results support extending the transdiagnostic stages of psychosis, anxiety, and depression to additional waves in ALSPAC (e.g., ages 24 and 26), potentially including linked data. Including participant information at later time-points can help cover a greater risk period for the onset of mental health symptoms and therefore stages 1b and 2. Finally, the results from our analyses may not generalise to those who were of non-white ethnicity and with evidence of social disadvantage due to patterns of missing data. Due to the likelihood of differential attrition in later waves in ALSPAC [36], our sample included fewer males and those with lower IQ. This may bias the observed relationships between Stage 1b psychosis and these risk factors. The proposed next steps in expanding these stage definitions using additional data could be augmented with longitudinal attrition weighting methods to improve generalisability.

Despite these limitations, this is the first study to devise data-informed transdiagnostic stages in a community sample of young adults followed from birth. Our findings are also supported by our robust approaches to handling missing data, and sensitivity analyses. Our proposed transdiagnostic stages could lead to future research to understand common risk factors, early shared neurobiological markers, pathways to the onset of mental disorders and to develop pragmatic risk prediction tools within ALSPAC and similar community-based cohorts. These could also help identify those young people at a higher risk of future mental ill-health associated with need for care. This could help develop recommendations for young people, their families, and clinicians supporting them to monitor for and prevent the onset of more serious mental health difficulties in adulthood.

## Supplementary information

Supplementary material
