# Supplementary material for: Empirically driven transdiagnostic stages in the development of mood, anxiety and psychotic symptoms in a cohort of youth followed from birth
Source: Transl Psychiatry. 2023 Mar 29;13:103. doi: 10.1038/s41398-023-02396-4 (PMC10052262; doi:10.1038/s41398-023-02396-4)
Supplement: Supplementary file 1 — Supplementary material [file 41398_2023_2396_MOESM1_ESM.docx]

# Supplementary Methods

## Operationalisation of stage 1b

We operationalised the transdiagnostic stages for the purposes of this study using the following principles: a) the overarching framework presented by Scott^1^, Shah ^2^ and colleagues, b) previous empirical research that pointed to stage specific markers for individual disorders and c) comparison of the observed prevalence of Stage 1b and 2 mental disorders in the Avon Longitudinal Study of Parents and Children (ALSPAC) with the expected prevalence of disorders and precursors in a general population sample. We specified that those with 1b level symptoms had either significant distress, or a functional impact associated with these symptoms directly indicating a need for care, and consistent with the general principles of Diagnostic and Statistical Manual for Mental Disorders (DSM) 5 and International Classification of Diseases (ICD) disorder definitions.

We adapted the staging model as presented by Scott, and Shah, using a consensus approach. This was necessary due to several reasons.

- Scott and Shah papers were salient conceptual advances for the field but were supported by limited prior empirical data. Previous studies with prospective data^3^ judged the inclusion of participants using clinical consensus for each participant resolving discrepant judgments in discussion among clinicians. This may not be feasible for most research studies where operational definitions are necessary. We also note other cohort studies^4, 5^ which attempt to test operational transdiagnostic frameworks aligned with the proposals from Shah and Scott. These studies utilise clinical help-seeking populations, where differential prevalence of different mental disorders (e.g., the 10-fold higher prevalence of anxiety compared with psychosis) can be accounted for by selective recruitment, or differences in help-seeking. Thus, existing recommendations for staging are based on limited evidence and entirely from clinical samples where selection biases may be prominent. They may also have limited international generalisability as they are reliant on different clinical service thresholds
- In translating clinical staging to a representative community-based sample such as ALSPAC, we expected several differences from clinical cohorts. First, the prevalence of depression and anxiety were likely to be substantially higher than that of psychosis or hypomania, based on their prevalence in the general population. Second, in community cohorts such as ALSPAC the presence of symptoms at most time-points were determined using screening measures (and rarely diagnostic instruments) rather than clinical impressions. We noted that crossing thresholds on a screening instrument alone may not be associated with a clinical diagnosis^6^ as the purpose of such instruments are often to screen out a disorder rather than establish diagnosis (case finding). In the ALSPAC dataset, even when diagnostic instruments were utilised (e.g., CIS-R at age 18 years), only 8.7% of participants meeting criteria for major depression had received this diagnosis (current or past) or were receiving treatment when this data were linked to a general practitioner treatment dataset^7^. While challenges to help-seeking are likely to explain this disparity, this also suggested the possibility that screening measures or diagnostic instruments may not translate fully to clinical assessments which have previously driven transdiagnostic staging^3^. Finally, indicators of treatment, cognitive impact of illness, or drops in functioning as Scott, Shah and others have suggested to be markers of stages were not available at points of clinical change in this cohort. We note that such data would not be expected to be available given that community cohort studies are not focused on acute changes in mental health presentations or their impact.
- In order to account for almost all eligible participants in ALSPAC using data available in community cohorts (unlike clinical help-seeking cohorts where recruitment can be selective, data collected during times of clinical change and clinical consensus obtained), we modified the stage definitions proposed by Scott, Shah and others in two ways.
  - Across all disorder definitions, a more stringent threshold was adopted to decrease the risk of false positives. We considered that the presence of severe depressive or anxiety symptoms were deemed to be 1b rather than Stage 2. Additionally, we specified the DSM criteria for a hypomanic episode as opposed to the use of a cut-off alone on the Hypomania Checklist-32. Similarly, we utilised the requirement for functional impact, distress or help-seeking to indicate the presence of significant psychotic symptoms. This meant that all Stage 1b symptoms were of likely clinical consequence. Although there may be some risk that some participants with later stages were included in our Stage 1b definition, this was reduced by our exclusion of participants with Stage 2 or more from further analyses of Stage 1b data.
  - We included the requirement for persistence or recurrence for ‘common mental disorders’ such as depression and anxiety in order to achieve a relatively similar prevalence as low prevalence mental health conditions such as hypomania and psychosis. First, this higher threshold reflects the difference in help-seeking for anxiety and depression (compared to that for psychosis or bipolar disorder) in clinical settings where staging is commonly applied. Second, this was necessary to explore associations and common risk factors across common and low prevalence mental health symptom stages with relatively similar power.
- Despite these differences, we remained consistent with the overall framework of staging and specifically 1b symptom stages where symptom severity and functional impact were accounted for across our stage definitions. We also note that these are the first operational definitions for translating clinical staging to a community-based cohort study, ensuring that the stage identification is rigorous. This will enable the next steps in examining other aspects of the staging model such as testing the hypothesized progression of stages, as suggested by the reviewer. Our results also suggest modifications for how staging as currently operationalised for clinical cohorts can be adapted to community cohorts.

The primary outcome was defined across ages 18 or 21 within the ALSPAC cohort. The use of two time-points in young adulthood allowed us to ensure that stage definitions were more rigorous, and to include hypomanic symptoms and bipolar mood instability along with outcome stages of psychosis and depression. We specified that a change or impairment in functioning was necessary across Stages 1b and 2 although these were operationalised differently for the two outcome stages. Stage 1b of psychotic or mood symptoms in young adulthood was determined at ages 18 or 21. This was determined based on the interview assessment conducted at age 18 as well as the questionnaire assessment completed at age 21 years.

### Stage 1b Psychosis

**Age 18.** We included the presence of 1 or more definite psychotic symptoms as rated by the interviewer that caused significant distress and which were clearly not attributed to sleep disturbance, fever or drug use. We specified that the symptoms should have occurred at least monthly in the preceding 6 months. Additionally, these symptoms were associated with a decline in peer, academic or vocational functioning, or led to professional help-seeking. The decline in functioning was rated by the young person as ‘much worse’ than the previous year during the same assessment session. This is adapted from the definition of ‘Psychotic Disorder’ previously utilised at this time point in the ALSPAC study ^8^.

**Age 21.** The presence of 1 or more self-reported definite psychotic symptoms (hearing voices, seeing visions and feeling suspicious) that caused significant distress and not attributable to sleep disturbance, cannabis or other drugs was included. Symptoms reported in the previous 6 months were included to capture participants who had developed new onset psychotic symptoms after the last assessment. Additionally, we specified that these young people should have had current impairment related to emotional symptoms at the same time point, based on three items from the Medical Outcomes Study Short Form 36 (SF-36)^9^. This was defined as the young person self-reporting that they accomplished less, was limited in the kind of work or activities they were able to do and completed tasks less carefully, all or most of the time in the previous month due to ‘emotional problems’.

### Stage 1b Hypomania

Stage 1b hypomania symptoms were determined at age 21 based on participants’ self-report on the Hypomania Checklist-32 item version (HCL-32^10^). This instrument has been validated extensively in clinical samples and more recently in non-clinical samples to screen for the presence of BD^11^. We used the previously accepted cut-score of 14 or more ^12, 13^ on the 32 item yes/no questionnaire referring to participants’ endorsement of hypomanic or related symptoms. In addition, we specified that participants must have endorsed an episode with such symptoms (i) lasting at least 4 days, (ii) associated with a negative impact on their family, social, vocational or leisure activities, and (iii) commented on by friends/family in either a positive or negative manner. These specifiers were also considered necessary to be aligned with the descriptors associated with DSM-IV and 5 (hypo)manic episodes. We considered an alternative definition excluding four items from the HCL 32 based on a Rasch analysis approach to this instrument^14^. This definition has recently been utilised in a report from ALSPAC as a measure of DSM-IV or 5 hypomanic episodes^15^. However, utilising this definition did not change the participants included in stage1b hypomania, probably because of the impact of the additional qualifiers.

### Stage 1b Depression

The presence of Stage 1b depressive symptoms was determined at ages 18 and 21 in line with that for psychosis. Two outcomes were selected to reflect Stage 1b depressive symptoms: (I) recurrent or persistent moderate depressive symptoms at both these time points and (II) severe depressive symptoms at either time point.

**Recurrent or persistent depressive symptoms** was considered to be present if depressive symptoms of a threshold severity were present at *both of the young adult time points (Age 18 and 21)*. Recurrent or persistent depressive symptoms ensured that we excluded those with mild and transient depressive symptoms as an outcome.

***Age 18.*** Data from the ALSPAC TF4 focus clinic included the computer assisted self-assessment for depressive symptoms based on the structured Clinical Interview Schedule- Revised (CIS-R^16^) instrument. International Classification of Diseases (ICD-10^17^) diagnostic criteria were applied to derive the presence of mild, moderate or severe depressive episodes using the number of depressive symptoms present and duration of depressive symptoms present. The presence of moderate depressive episode as per ICD-10 criteria was selected to mark the presence of Stage 1b depressive symptoms at this point, as it was considered that such symptoms were likely to be associated functional impairment in line with ICD-10 guidelines for moderate depression.

***Age 21.*** At this time point, depressive symptoms were assessed using self-report on the SMFQ regarding depressive symptoms in the previous 2 weeks. A cut-score of 11 or more on this scale was chosen to indicate the presence of Stage 1b depressive symptoms at this time point as this was associated with reasonable sensitivity and specificity for a depressive episode within the ALSPAC cohort at age 18^18^. In order to ensure significant functional impact necessary for this stage, we specified that these young adults should have had recent functional impairment due to emotional symptoms in a similar time period along with the presence of such depressive symptoms. This was based on the previously mentioned three items from SF36 where young adults reporting that they had accomplished less, were limited in their activities, or were less careful in their activities (all or most of the time) due to emotional problems in the preceding month.


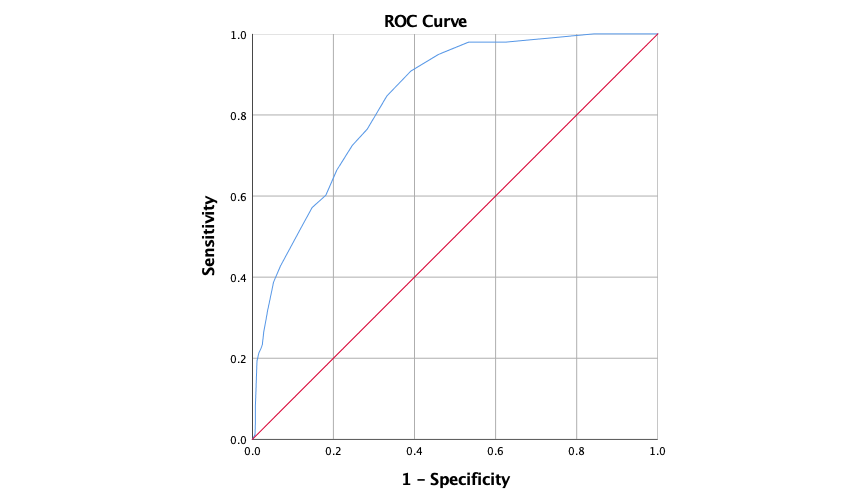


Supplementary Figure 1. Receiver Operating Characteristic Curve examining the relationship between Age 18 sMFQ scores and Age 18 ICD-10 depression (outcome), AUC=0.838, SE=0.018, p<0.001

**Severe depressive symptoms with functional impairment** were considered to be present if such symptoms and impairment were present at *either of the young adult time points*. This is because the presence of severe depressive symptoms even at one time point is likely to require psychological and pharmacological interventions as would be required for significant manic or psychotic symptoms.

***Age 18.*** We selected young adults who self-reported depressive symptoms on the CIS-R at a severe level as indicated by ICD-10 criteria. This included the requirement for three of the primary depressive symptoms and at least five secondary depressive symptoms. Given the disorder level validation and severity cut-off associated with CIS-R definitions, additional specifiers were not considered necessary.

***Age 21.*** Severe depressive symptoms at this time point was determined using participants’ self-report on the sMFQ. A cut-score of 20 or more was selected to indicate the presence of severe depressive symptoms as outlined below. In addition, we specified that young adults should have reported additional functional impairment at this time. As described earlier, the young adults should have reported that they had accomplished less, were limited in their activities, or were less careful in their activities, all or most of the time due to emotional problems in the preceding month at the same timepoint.

As pre-specified cut-scores for symptom severity levels were not available for sMFQ, we aimed to identify optimal cut-scores from the data available from sMFQ and CIS-R derived depression diagnoses completed at the same sitting at age 18. This is in line with previous examinations of optimal cut-scores on sMFQ within the ALSPAC cohort^18^. We performed Receiver-Operating-Characteristic (ROC) curves for the total sMFQ score against an outcome of Severe depressive episode on CIS-R and examined the sensitivity, specificity and predictive values to identify the optimal cut-point. A cut-score of 20 was selected as this score was associated with the highest positive predictive value (55.2%) and reasonable negative predictive values for a severe depressive episode (**STable 2**).

**Supplementary Table 1.** Predictive properties of sMFQ for ICD-10 Severe depression (n=45) in subset of those with MDD in the ALSPAC cohort at age 18 (n=195) [AUC= 0.68, SE= 0.05, p<0.001]

| **Cut-point**  **(value or more)** | **17** | **18** | **19** | **20** | **21** |
| --- | --- | --- | --- | --- | --- |
| Sensitivity (%) | 62.2 | 57.8 | 51.1 | 46.7 | 35.6 |
| Specificity (%) | 71.9 | 76.5 | 80.8 | 83.8 | 87.7 |
| Prevalence (%) | 14.4 | 14.4 | 14.4 | 14.4 | 14.4 |
| PPV (%) | 46.2 | 49.7 | 52.1 | 55.2 | 53.8 |
| NPV (%) | 91.2 | 91.5 | 90.8 | 90.3 | 89.0 |

We further validated the cut-off of 20 or more as a valid threshold for severe MDD by examining the derived prevalence of severe MDD among those with MDD at Age 21. Using a cut-score of 20, along with the impairment criterion specified above, we identified 56 cases with severe MDD out of a total of 387 at age 21. At age 18, the prevalence of ICD-10 severe depression was 14.4% of those with MDD suggesting a similar prevalence to that identified at age 21 (14.5%). Furthermore, a cut score of 20 was associated with the threshold for severe depression determined using a structured instrument in a recent clinical trial in young people with MDD^19^.

### Stage 1b Anxiety

While the initial cross-cutting conceptualisation of staging covered severe mood and psychotic disorders^1^, later transdiagnostic models for youth mental health disorders have also included anxiety disorders ^2^. Therefore, we attempted to address the role of anxiety symptoms in the progression of mood and psychotic disorder stages. We conceptualised Stage 1b anxiety to be similar to that of depression, i.e. recurrent or persistent moderate anxiety at both time points or severe and impairing anxiety at either age 18 or 21 (Stage 1b anxiety considered as present if either definitions were met).

**Recurrent or persistent moderate anxiety** was considered present if clinically significant symptomatic anxiety was present at both ages 18 and 21 years.

***Age 18.*** We included those with a score of 2 or more on either the overall anxiety or the phobic anxiety ratings using the Clinical Interview Schedule- Revised (CIS-R^16^) consistent with previously used thresholds for this instrument ^16, 20^.

***Age 21.*** We considered a score of 10 or more on the Generalised Anxiety Disorder-7 item scale ^21^ to indicate the presence of moderate anxiety, consistent with the recommendations for this instrument.

**Severe and impairing anxiety** at either time points was considered to be present at ages 18 or 21 if either of the following self-report ratings were positive.

***Age 18.*** Scores on either the anxiety rating, which refers to more generalised anxiety or the phobia symptom rating, which refers to phobic anxiety using the Clinical Interview Schedule- Revised (CIS-R^16^) was used. At the usually recommended cut-score of 2, there may not be a significant impairment consistent with the other definitions required for this stage. Therefore, we selected a higher cut-off of 3 for these variables. At this prevalence, we also found that the observed prevalence of anxiety matched the expected prevalence of severe and impairing anxiety (associated with phobias and generalised anxiety, 4.9%) in epidemiological data in this age group^22^.

***Age 21.*** We included those with a score of 10 or more on the Generalised Anxiety Disorder- 7 item scale but in addition, required that they had recent functional impairment due to emotional symptoms. This was based on the aforementioned three items from SF-36 relating to young people accomplishing less, being limited in their activities, or completing tasks less carefully, all or most of the time due to emotional problems in the preceding month.

Given the expected higher prevalence of 1b anxiety compared to other disorders, we constructed a definition of ‘higher threshold Stage 1b anxiety’ to create a Stage definition which was likely to be similar in prevalence to that of depression, psychosis and hypomania. In this definition, recurrent or persistent anxiety was defined in the same manner, but severe and impairing anxiety was determined based on a higher symptom threshold. At age 18, we used a higher CIS-R rating of 4 or higher and at age 21 we utilised a higher threshold on GAD-7 (≥15) combined with concurrent impairment in functioning all or most of the time, again on the same SF-36 items.

To ensure that we captured symptoms at a Stage 1b level, we excluded those with a higher symptom stage (Stage 2 or more, or 2+) at these time points. The operationalisation of these stages are described further below.

## Operationalisation of stage 2+

We defined Stage 2 level psychotic, mood, or anxiety problems as the presence of severe depression with impairment, threshold psychosis with impairment, a lifetime manic episode, hypomania with moderate to severe depressive episodes which were in turn associated with impairment, or severe anxiety with impairment. Detailed disorder specific stage definitions can be found below.

### Stage 2+ Psychosis

Threshold psychosis was operationalised using the cut-offs modified from the Comprehensive Assessment of At-Risk Mental States (CAARMS^23^) as this is widely accepted as a significant level of psychosis requiring intervention. However, as the frequencies available in ALSPAC (weekly, daily) did not correspond to those required for CAARMS ratings (weekly, 3-6 times a week, daily), the 3-6 times a week category was approximated to ‘most days’. Thus, the Stage 2 definition in ALSPAC corresponds to clear psychotic symptoms, as determined by a trained rater which was present most days, at least one hour or more at a time, associated with considerable distress and at least some functional decline compared to the previous year. The decline in functioning was rated by the young person as much worse than the previous year during the same assessment session. Such criteria could only be defined at age 18 as sufficient data to determine threshold level psychotic symptoms was only available at this time point.

### Stage 2+ Depression

Presence of Stage 2 level depression was determined at ages 18 and 21 combining the presence of severe depressive symptoms as well as functional impairment.

**Age 18.** The presence of severe depression was established using the young person’s rating on the computer assisted interview using the CIS-R instrument as described previously. In addition, we required that young people must have had functional impairment defined as a decline in functioning compared to the previous year, as with the criteria for threshold psychotic symptoms at this time point.

**Age 21.** A young person’s rating of their mood on the s-MFQ at a threshold of 20 or over was indicative of them having severe depressive symptoms, as outlined previously. At the same time, we specified that these young people must have had significant impairment due to emotional symptoms. This was based on SF-36 items where the young adults reported that they had accomplished less, were limited in their activities, or were less careful in their activities, all of the time (more persistent than in Stage 1b) due to emotional problems in the preceding month at the same timepoint.

### Stage 2+ Bipolar

Presence of Stage 2 level bipolar mood syndromes was determined across ages 18 and 21 combining the presence of moderate to severe depressive symptoms as well as functional impairment at either time point, and the presence of threshold manic or hypomanic episodes at age 21. Two outcomes were selected to reflect Stage 1b bipolar symptoms (a) presence of an equivalent lifetime manic episode as per a diagnosis of bipolar type I and (b) presence of Stage 1b hypomania in addition to a significant depressive episode at either age 18 or 21.

**Bipolar I.** Presence of self-reported lifetime manic episodes was determined using the young people’s rating on the HCL-32. Young people needed to endorse 14 or more hypomanic symptoms, lasting one week or more, associated with negative impact on their functioning, and which significant others commented on in a negative manner. This is consistent with the DSM definition of mania. Similarly in a recent study of bipolar high-risk youth and control participants, only the high-risk group endorsed symptoms lasting a week or more on the HCL-32, while the control participants reported symptoms lasting less than a week indicating the validity of this duration cut-off ^24^.

**Bipolar II.** In addition to a lifetime hypomanic episode as defined in Stage 1b hypomania, we specified that young people must have fulfilled criteria for moderate to severe depressive episodes at either age 18 or 21 years. At age 18, young people reporting moderate or severe depressive episodes on CIS-R were included. At age 21, young people reporting current depressive symptoms associated with a cut-score of 11 or more on the sMFQ, associated with recent functional impairment due to emotional symptoms were included. This was based on the young adults reporting that they had accomplished less, were limited in their activities, or were less careful in their activities, all or most of the time due to emotional problems in the preceding month at the same timepoint.

### Stage 2+ Anxiety

Presence of Stage 2 level anxiety was defined at ages 18 and 21 years combining the presence of severe anxiety symptoms and functional impairment, in line with the definition for Stage 2 depression.

**Age 18.** We required that young people should have a higher threshold of anxiety symptoms on CIS-r anxiety and phobia ratings, equal to 4 or more in severity, along with an impairment in functioning compared to the previous year.

**Age 21.** We specified that young people should have a score of 15 or more on GAD-7 scale indicating severe anxiety, along with pervasive functional impairment (all of the time, more persistent than in Stage 1b) due to emotional symptoms, as defined by the items on SF-36 specified above.

## Risk factors

We explored a number of prior factors as predictors of Stage 1b disorder. This was to examine whether Stage 1b disorders shared common risk factors. Risk factors included:

**Sex at birth.** Sex as specified at birth was chosen as opposed to self-identified gender due to the effect of sex at birth on early life characteristics such as obstetric complications.

**Ethnicity and social class reported during pregnancy**. The child’s ethnicity was determined based on mother’s and father’s self-reported ethnicity and categorised as ‘Caucasian’ or ‘Non-Caucasian’. Social class was determined based on mother or partner’s occupation^25^. The higher social class amongst the two parents were selected in line with previous determination of this variable^26^.

**Obstetric complications.** While several obstetric complications were associated with later life psychotic symptoms in previous reports from the ALSPAC study^27^, resuscitation status at birth was utilised as a single marker of several pathophysiological processes associated with perinatal insults. The 5-minute Apgar score was utilised as a second marker for sensitivity analyses.

**First degree family history of mental disorders.** The presence of family history was collected from parents’ self-report at several time points including pregnancy, and when the child was aged 8 and 12 years. For the father, the data was only included if the partner variables were completed by the young person’s biological father. Family history of schizophrenia, and that of severe depression were considered for inclusion in the models below.

**Early life psychosocial adversity.** The presence of psychosocial adversity was modelled in this study using the Family Adversity Index^28^ developed from the ALSPAC data. This composite index covers several family-based risk factors such as mother’s age, housing, educational status, financial status, partner relationship, family characteristics, social network, maternal psychopathology, social networks, and crime. Adversity in early life (child at 2-4 years) was measured using the ‘short index’ which includes 15 items rated as 0 or 1. A ‘long index’ which includes 18 variables measured during pregnancy was used for sensitivity analyses.

**Emotional and Behavioral difficulties in early adolescence:** We used the Strengths and Difficulties Questionnaire^29^ (SDQ) completed at age 10 by the participant’s parent. The subscale scores on emotional problems, peer difficulties, hyperactivity and conduct problems were summated to obtain a composite marker of emotional and behavioural difficulties^29^ (EBD). Those scoring below the median difficulties score on the EBD subscales of SDQ has been previously utilised as a marker of resilience in previous studies^30^ including the ALSPAC cohort^31^.

**Alcohol use.** the severity of alcohol use was determined using the total score on the Alcohol Use Disorders Identification Test (AUDIT^32^) at age 16. Those with a score of 8 or more were considered to have at least a hazardous level of drinking^33^.

**Cannabis use.** The primary measure of cannabis use was young people’s report at age 16 on whether they had ever used cannabis. Cannabis use was also measured by the participants’ self-report on the Cannabis Abuse Screening Test^34^. The total score on the six-item scale was utilised to identify problematic substance use.

**Negative life events.** Negative life events have been associated with the onset of several mental health conditions, particularly depression, in prospective community studies ^35^. Conversely, those with higher mental health symptoms have been noted to have greater risk of subsequent life events among those with depression^36^ and those at risk for psychosis^37^. The presence of negative life events between the ages of 12 and 16 was determined based on adolescents’ self-report which covered parental relationships, peer relationships, difficulties at school, and losses ^38^. We utilised a dichotomous variable indicating the presence or absence of negative life events that were self-rated by young people as being ‘highly unpleasant’. A second variable where a lower rating of ‘unpleasant’ life events was selected for sensitivity analysis.

**Analysis**

**Networks:** Further details on the implementation of the regression based Ising model: The Ising model uses regularised logistic regression which shrinks unimportant parameters to zero, thereby returning an accurately sparse network. Fitting the Ising model requires specifying two tuning parameters: gamma, controlling the degree to which sparse networks are preferred over dense networks, and the rule for determining the presence or absence of an edge, either an AND-rule (both coefficients are non-zero) or an OR-rule (at least one coefficient is non-zero). A validation study showed that the model has excellent specificity and good sensitivity for detecting non-zero edge-weights and that sensitivity can be markedly improved by relaxing tuning parameters with little effect on specificity ^39^. Consequently, to maximise our sensitivity for detecting edge-weights, we chose to use a gamma of zero (indicating no penalty for more connections) combined with an OR-rule.

**Factor analyses**: An exploratory factor analyses (Mplus v8.0, Model: type= efa (1,2) suggested that only one factor may be specified. Therefore, a confirmatory factor analysis (CFA) was specified with the four 1b stages as indicator variables and a maximum likelihood estimator with a probit link function (Weighted Least Square Means and Variance Adjusted, WLSMV) among those with complete cases.

**Imputation Implementation:** Where data underlying dichotomous prior risk variables was continuous, we used these continuous measures in the imputation model and derived dichotomous risk variables for the purpose of regression subsequent to imputation. These variables were FAI (short index, age 8), AUDIT total score (age 16), and EBD score of the SDQ (age 10). In addition to prior risk variables, we used a set of 28 auxiliary variables to assist with multiple imputation. Variables were chosen for their level of completeness and ability to predict prior risk variables. Imputation of all variables was conducted using the random forest algorithm implemented in the ‘mice’ function of the mice package in R.

Analyses codes are available upon request.

# Supplementary Results

**Supplementary Fig 2:** Participants included and data available at various time points

**Supplementary Table 2.** Descriptive statistics for demographic, risk, and clinical variables, including Stage 1b variables, stratified by whether or not an individual had complete Stage 1b data (data on all Stage 1b disorders), partial Stage 1b data (data on at least one Stage 1b disorder), or missing Stage 1b data (no data on any Stage 1b disorders).

|  | **Complete (N=3346)** | **Partial (N=2326)** | **Missing (N=9973)** | **Overall (N=15645)** |
| --- | --- | --- | --- | --- |
| **Sex** |  |  |  |  |
| male | 1182 (35.4%) | 1183 (50.9%) | 5327 (56.8%) | 7692 (51.1%) |
| female | 2160 (64.6%) | 1141 (49.1%) | 4047 (43.2%) | 7348 (48.9%) |
| Missing | <5 (<1%) | <5 (<1%) | 599 (6.0%) | 605 (3.9%) |
| **Ethnicity** |  |  |  |  |
| caucasian | 2954 (96.0%) | 1923 (95.5%) | 6647 (94.4%) | 11524 (94.9%) |
| non-caucasian | 124 (4.03%) | 91 (4.52%) | 398 (5.65%) | 613 (5.05%) |
| Missing | 268 (8.0%) | 312 (13.4%) | 2928 (29.4%) | 3508 (22.4%) |
| **Resuscitated at birth** |  |  |  |  |
| no | 1321 (70.9%) | 929 (71.2%) | 3300 (68.9%) | 5550 (69.8%) |
| yes | 541 (29.1%) | 375 (28.8%) | 1489 (31.1%) | 2405 (30.2%) |
| Missing | 1484 (44.4%) | 1022 (43.9%) | 5184 (52.0%) | 7690 (49.2%) |
| **Family history (severe depression)** |  |  |  |  |
| no | 2645 (80.4%) | 1736 (79.6%) | 6229 (80.4%) | 10610 (80.3%) |
| yes | 645 (19.6%) | 445 (20.4%) | 1514 (19.6%) | 2604 (19.7%) |
| Missing | 56 (1.7%) | 145 (6.2%) | 2230 (22.4%) | 2431 (15.5%) |
| **Family history (schizophrenia)** |  |  |  |  |
| no | 3279 (99.7%) | 2175 (99.5%) | 7724 (99.7%) | 13178 (99.7%) |
| yes | 11 (0.334%) | 10 (0.458%) | 20 (0.258%) | 41 (0.310%) |
| Missing | 56 (1.7%) | 141 (6.1%) | 2229 (22.4%) | 2426 (15.5%) |
| **Harmful drinking (Age 16)** |  |  |  |  |
| no | 1515 (64.8%) | 615 (56.9%) | 482 (54.3%) | 2612 (60.7%) |
| yes | 822 (35.2%) | 465 (43.1%) | 405 (45.7%) | 1692 (39.3%) |
| Missing | 1009 (30.2%) | 1246 (53.6%) | 9086 (91.1%) | 11341 (72.5%) |
| **Cannabis use (ever; Age 16)** |  |  |  |  |
| no | 2021 (74.2%) | 845 (67.4%) | 761 (69.8%) | 3627 (71.6%) |
| yes | 701 (25.8%) | 409 (32.6%) | 330 (30.2%) | 1440 (28.4%) |
| Missing | 624 (18.6%) | 1072 (46.1%) | 8882 (89.1%) | 10578 (67.6%) |
| **Highly unpleasant life events** |  |  |  |  |
| no | 1064 (38.9%) | 520 (41.1%) | 419 (37.7%) | 2003 (39.2%) |
| yes | 1671 (61.1%) | 746 (58.9%) | 693 (62.3%) | 3110 (60.8%) |
| Missing | 611 (18.3%) | 1060 (45.6%) | 8861 (88.8%) | 10532 (67.3%) |
| **WISC (FSIQ)** |  |  |  |  |
| Mean (SD) | 108 (16.0) | 105 (16.1) | 99.3 (16.0) | 104 (16.5) |
| Median [Min, Max] | 108 [45.0, 149] | 105 [48.0, 146] | 99.0 [46.0, 151] | 104 [45.0, 151] |
| Missing | 650 (19.4%) | 540 (23.2%) | 7109 (71.3%) | 8299 (53.0%) |

## Risk factors for specific stage 1b disorders

Below are tables describing missing data in prior risk variables (**Supplementary** **Table 3**) and correlations between prior risk factors (**Supplementary Table 4**), as well as the results of logistic regressions of each Stage 1b disorder onto each risk factor without imputation. **Supplementary Table 5** describes the results for those with Stage 1b data for all disorders, with variable missingness on risk factors (N= 1817- 3265) which were dealt with using pair-wise deletion of cases. **Supplementary Table 4** includes the results using all available data for each Stage 1b disorder (N=3097- 5545) and pair-wise deletion of cases for each analysis separately. Results mirror those in the main text suggesting that the imputed datasets are accurately representing missing cases or at least accurately replicating existing data.

**Supplementary Table 3.** Counts (percentage) of missing data for each prior risk factor.

| **Prior risk factor** | **Missing Data Count (%)** |
| --- | --- |
| Sex at birth (female) | 4 (0.12%) |
| Resuscitated at birth | 1452 (44.42%) |
| Child ethnic background | 263 (8.05%) |
| Family social class (Class I or II) | 280 (8.57%) |
| FAI risk category at age 4 | 857 (26.22%) |
| Family history of depression | 56 (1.71%) |
| Emotional and behavioral difficulties | 961 (29.40%) |
| Cannabis use | 603 (18.45%) |
| Hazardous drinking, age 16 | 980 (29.98%) |
| Highly unpleasant life events | 591 (18.08%) |
| Full Scale IQ | 634 (19.39%) |

Notes: FAI- Family Adversity Index; IQ- Intelligence Quotient

**Supplementary Table 4.** Tetrachoric and biserial correlations between prior risk factors. Pairwise deletion of cases was used to handle missing data.

| **Prior risk variable** | **1 .** | **2.** | **3.** | **4.** | **5.** | **6.** | **7.** | **8.** | **9.** | **10.** | **11.** |
| --- | --- | --- | --- | --- | --- | --- | --- | --- | --- | --- | --- |
| 1. Sex at birth (female) | 1.00 |  |  |  |  |  |  |  |  |  |  |
| 2. Resuscitated at birth | -0.05 | 1.00 |  |  |  |  |  |  |  |  |  |
| 3. Child ethnic background | 0.01 | -0.01 | 1.00 |  |  |  |  |  |  |  |  |
| 4. Family social class (Class I or II) | -0.07 | 0.05 | -0.06 | 1.00 |  |  |  |  |  |  |  |
| 5. FAI risk category at age 4 | 0.00 | 0.00 | 0.17 | -0.12 | 1.00 |  |  |  |  |  |  |
| 6. Family history of depression | 0.04 | 0.00 | -0.01 | -0.11 | 0.30 | 1.00 |  |  |  |  |  |
| 7. Emotional and behavioral difficulties | -0.07 | -0.03 | -0.03 | -0.11 | 0.15 | 0.24 | 1.00 |  |  |  |  |
| 8. Cannabis use | 0.07 | 0.02 | 0.11 | 0.01 | 0.13 | 0.10 | 0.05 | 1.00 |  |  |  |
| 9. Hazardous drinking, age 16 | 0.03 | -0.02 | 0.12 | 0.03 | 0.09 | 0.02 | -0.05 | **0.64** | 1.00 |  |  |
| 10. Highly unpleasant life events | **0.28** | -0.07 | 0.11 | -0.04 | 0.08 | 0.14 | 0.10 | 0.07 | 0.09 | 1.00 |  |
| 11. Full Scale IQ | -0.12 | 0.05 | 0.03 | 0.32 | -0.05 | -0.07 | -0.20 | 0.12 | 0.03 | -0.06 | 1.00 |

Notes: FAI- Family Adversity Index; IQ- Intelligence Quotient

**Supplementary Table 5.** Odds-ratios and associated 95% confidence intervals from separate logistic regressions of disorder specific Stage 1b categories onto prior risk factors. Sample is those with complete data on all Stage 1b disorders (N= 1817- 3265), with no imputation of risk variables and pair-wise deletion of cases with missingness on risk factors.

| **Prior Risk Variable** | **Depression** | | **Hypomania** | | **Psychosis** | | **Anxiety** | | **Anxiety HT** | |
| --- | --- | --- | --- | --- | --- | --- | --- | --- | --- | --- |
|  | **N** | **OR (95% CI)** | **N** | **OR (95% CI)** | **N** | **OR (95% CI)** | **N** | **OR (95% CI)** | **N** | **OR (95% CI)** |
| Sex at birth (female) | **3265** | **2.56 (1.30, 5.63)** | 3265 | 1.25 (0.65, 2.57) | **3265** | **4.48 (1.56, 18.87)** | **3265** | **2.02 (1.46, 2.87)** | **3265** | **2.54 (1.57, 4.33)** |
| Resuscitated at birth | 1817 | 0.89 (0.37, 1.93) | 1817 | 1.14 (0.43, 2.73) | 1817 | 0.89 (0.25, 2.61) | 1817 | 0.81 (0.53, 1.22) | 1817 | 0.98 (0.55, 1.68) |
| Child ethnic background | 3006 | 1.16 (0.19, 3.82) | 3006 | 2.46 (0.58, 7.04) | 3006 | 1.06 (0.06, 5.07) | 3006 | 1.14 (0.50, 2.23) | 3006 | 0.84 (0.20, 2.29) |
| Family social class (Class I or II) | 2989 | 0.86 (0.47, 1.59) | 2989 | 1.98 (0.96, 4.49) | 2989 | 0.53 (0.24, 1.17) | 2989 | 1.07 (0.78, 1.46) | 2989 | 0.97 (0.64, 1.48) |
| FAI risk category at age 4 | 2412 | 1.39 (0.71, 2.81) | 2412 | 1.11 (0.48, 2.64) | 2412 | 1.04 (0.40, 2.78) | **2412** | **1.75 (1.22, 2.52)** | **2412** | **2.20 (1.33, 3.77)** |
| Family history of depression | 3213 | 1.48 (0.75, 2.72) | 3213 | 0.50 (0.15, 1.27) | 3213 | 2.10 (0.90, 4.59) | **3213** | **1.99 (1.45, 2.71)** | **3213** | **2.28 (1.50, 3.43)** |
| Emotional and behavioral difficulties | **2308** | **2.89 (1.43, 6.19)** | 2308 | 1.36 (0.58, 3.20) | **2308** | **3.29 (1.22, 10.38)** | **2308** | **2.04 (1.43, 2.92)** | **2308** | **3.56 (2.12, 6.24)** |
| Cannabis use | 2666 | 1.63 (0.84, 3.05) | 2666 | 1.17 (0.48, 2.57) | 2666 | 1.17 (0.41, 2.88) | 2666 | 1.35 (0.95, 1.90) | 2666 | 1.25 (0.77, 1.98) |
| Hazardous drinking, age 16 | 2289 | 1.22 (0.62, 2.33) | 2289 | 1.72 (0.74, 3.93) | 2289 | 1.87 (0.73, 4.81) | 2289 | 1.35 (0.95, 1.90) | 2289 | 1.38 (0.87, 2.17) |
| Highly unpleasant life events | **2678** | **2.41 (1.20, 5.37)** | 2678 | 1.17 (0.55, 2.64) | **2678** | **13.13 (2.73, 235.82)** | **2678** | **1.89 (1.33, 2.74)** | **2678** | **2.38 (1.45, 4.09)** |
| FSIQ (WISC) | 2635 | 0.99 (0.98, 1.01) | 2635 | 1.01 (0.99, 1.04) | **2635** | **0.97 (0.95, 1.00)** | 2635 | 0.99 (0.98, 1.00) | **2635** | **0.98 (0.97, 0.99)** |

**Supplementary Table 6.** Odds-ratios and associated 95% confidence intervals from separate logistic regressions of disorder specific Stage 1b categories onto prior risk factors (N=3097- 5545) . No imputation, pair-wise deletion of cases.

| **Prior Risk Variable** | **Depression** | | **Hypomania** | | **Psychosis** | | **Anxiety** | | **Anxiety HT** | |
| --- | --- | --- | --- | --- | --- | --- | --- | --- | --- | --- |
|  | **N** | **OR (95% CI)** | **N** | **OR (95% CI)** | **N** | **OR (95% CI)** | **N** | **OR (95% CI)** | **N** | **OR (95% CI)** |
| Sex at birth (female) | **5545** | **2.87 (1.61, 5.52)** | 3276 | 1.25 (0.65, 2.57) | **5639** | **3.75 (1.85, 8.65)** | **5539** | **1.99 (1.54, 2.59)** | **5542** | **2.64 (1.78, 4.04)** |
| Resuscitated at birth | 3097 | 0.88 (0.40, 1.75) | 1825 | 1.15 (0.44, 2.73) | 3155 | 0.72 (0.28, 1.59) | 3088 | 0.77 (0.53, 1.07) | 3089 | 0.89 (0.54, 1.43) |
| Child ethnic background | 4983 | 1.34 (0.32, 3.67) | 3015 | 2.47 (0.59, 7.06) | 5059 | 1.14 (0.18, 3.74) | 4979 | 1.20 (0.64, 2.05) | 4982 | 1.25 (0.48, 2.64) |
| Family social class (Class I or II) | 4965 | 0.88 (0.52, 1.51) | 2999 | 1.99 (0.96, 4.51) | 5038 | 0.87 (0.48, 1.62) | 4961 | 1.08 (0.84, 1.40) | 4964 | 1.09 (0.76, 1.57) |
| FAI risk category at age 4 | 4069 | 1.23 (0.66, 2.33) | 2418 | 1.11 (0.48, 2.63) | 4125 | 1.47 (0.68, 3.34) | **4062** | **1.45 (1.08, 1.95)** | **4064** | **1.85 (1.20, 2.93)** |
| Family history of depression | 5352 | 1.60 (0.90, 2.73) | 3223 | 0.50 (0.15, 1.26) | **5437** | **2.33 (1.25, 4.19)** | **5348** | **1.98 (1.52, 2.55)** | **5351** | **2.29 (1.58, 3.27)** |
| Emotional and behavioral difficulties | **3743** | **2.76 (1.43, 5.66)** | 2313 | 1.36 (0.58, 3.19) | **3786** | **2.90 (1.31, 7.03)** | **3737** | **2.06 (1.52, 2.79)** | **3739** | **3.62 (2.24, 6.11)** |
| Cannabis use | 3898 | 1.72 (0.95, 3.06) | 2670 | 1.17 (0.48, 2.57) | 3916 | 1.56 (0.74, 3.15) | 3900 | 1.13 (0.83, 1.53) | 3902 | 1.13 (0.72, 1.71) |
| Hazardous drinking, age 16 | 3349 | 1.39 (0.76, 2.53) | 2291 | 1.71 (0.74, 3.92) | 3365 | 2.07 (0.97, 4.54) | 3353 | 1.18 (0.87, 1.60) | 3354 | 1.33 (0.87, 2.01) |
| Highly unpleasant life events | **3922** | **2.98 (1.51, 6.57)** | 2682 | 1.17 (0.55, 2.64) | **3940** | **10.38 (3.14, 64.17)** | **3924** | **2.23 (1.62, 3.12)** | **3926** | **2.69 (1.69, 4.47)** |
| FSIQ (WISC) | 4390 | 1.00 (0.98, 1.01) | 2642 | 1.01 (0.99, 1.04) | **4455** | **0.98 (0.96, 1.00)** | **4387** | **0.99 (0.98, 1.00)** | **4390** | **0.99 (0.97, 1.00)** |

## Confirmatory Factor Analysis

MODEL FIT INFORMATION

Number of Free Parameters 8

Chi-Square Test of Model Fit, 1.998 (df= 2), p= 0.3683

RMSEA (Root Mean Square Error Of Approximation): Estimate= 0.000 (90% CI, 0.000, 0.035), Probability RMSEA <= .05 =0.998)

CFI/TLI= 1.000, SRMR (Standardized Root Mean Square Residual)= 0.064

MODEL RESULTS

Two-Tailed

Estimate S.E. Est./S.E. P-Value

LATENT1 BY

S1B_DEP 1.000 0.000 999.000 999.000

S1B_HYP -0.039 0.095 -0.409 0.682

S1B_PSY 0.643 0.098 6.567 0.000

S1B_ANX 0.872 0.152 5.733 0.000

Thresholds

S1B_DEP$1 2.162 0.056 38.795 0.000

S1B_HYP$1 2.259 0.061 36.975 0.000

S1B_PSY$1 2.397 0.070 34.139 0.000

S1B_ANX$1 1.537 0.034 44.570 0.000

Variances

LATENT1 0.927 0.161 5.756 0.000

IRT PARAMETERIZATION

Item Discriminations

LATENT1 BY

S1B_DEP 3.567 4.252 0.839 0.402

S1B_HYP -0.038 0.092 -0.409 0.682

S1B_PSY 0.788 0.158 4.979 0.000

S1B_ANX 1.548 0.490 3.158 0.002

Item Difficulties

S1B_DEP$1 2.246 0.208 10.785 0.000

S1B_HYP$1 -60.184 146.941 -0.410 0.682

S1B_PSY$1 3.874 0.511 7.578 0.000

S1B_ANX$1 1.830 0.178 10.287 0.000

Variances

LATENT1 1.000 0.000 0.000 1.000

R-SQUARE

Observed Residual

Variable Estimate Variance

S1B_DEP 0.927 0.073

S1B_HYP 0.001 0.999

S1B_PSY 0.383 0.617

S1B_ANX 0.706 0.294

## Network Diagnostics

Using the *bootnet* package in R, we tested the accuracy and stability of edge-weights and intercepts from the model by generating 1000 non-parametric, boot-strapped samples and using these to (a) generate confidence intervals for parameters and (b) examine the effect of case dropping on parameter estimates. Boot-strapped difference tests showed that the only two edges reliably different from one another were the edges between anxiety and psychosis and depression and anxiety, with the edge between depression and anxiety proving reliably stronger (**Supplementary Figure 3**). Difference tests are not computed between present and absent edges as the presence versus absence of an edge is already assayed via the regularisation component of the Ising model. Correlations between parameters from boot-strapped samples and the original sample were high (**Supplementary Figure 4**), even with only 30% of the sample re-sampled, and Correlation Stability (CS) coefficients were adequate (CS_intercept_ = .75; CS_edge_ = .75; CS indicate maximum proportion of cases able to be dropped to retain a correlation of .7 between boot-strapped and original samples in 95% of samples), indicating that in general parameter estimates were highly stable.

| **A**  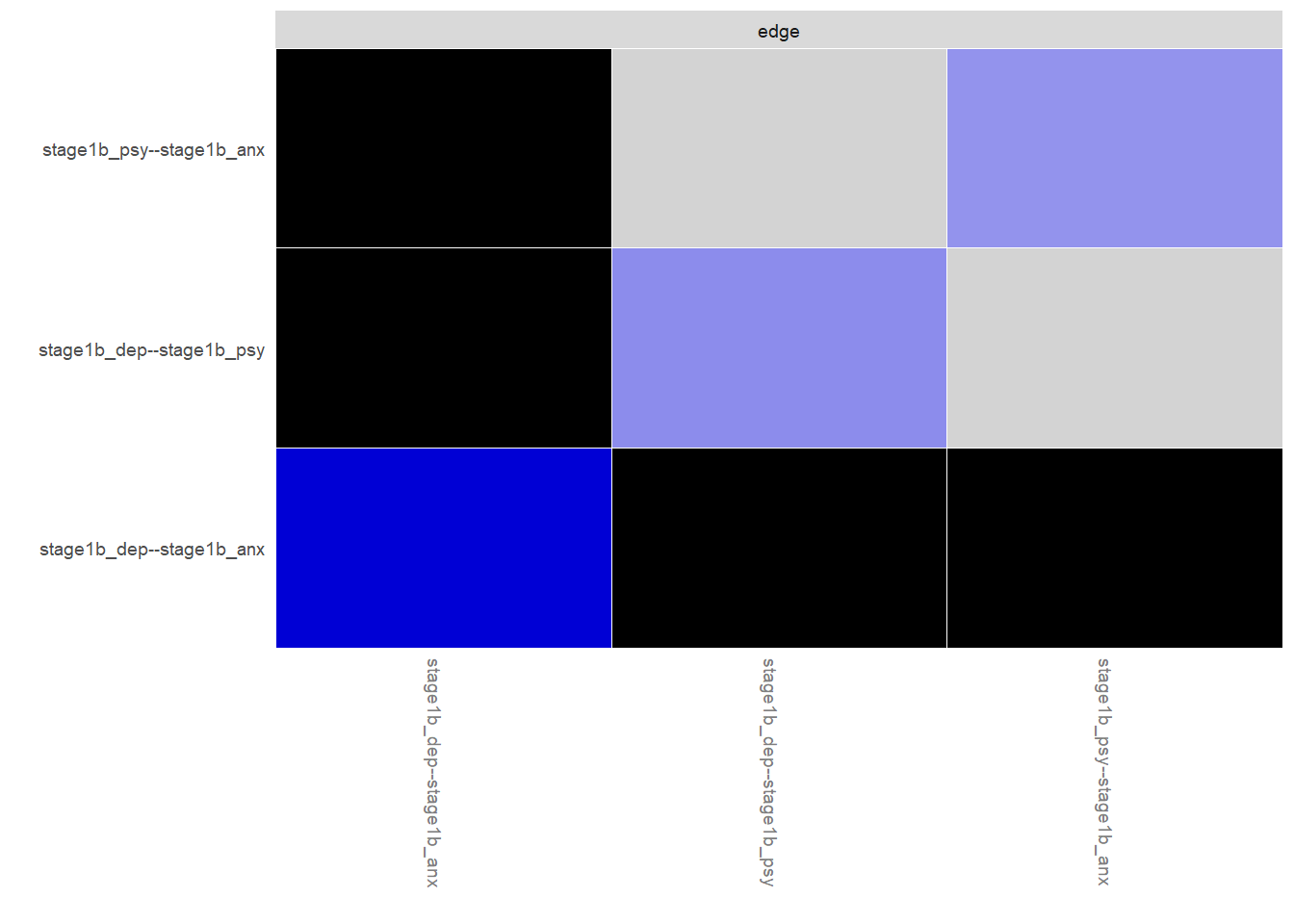 |
| --- |
| **B** 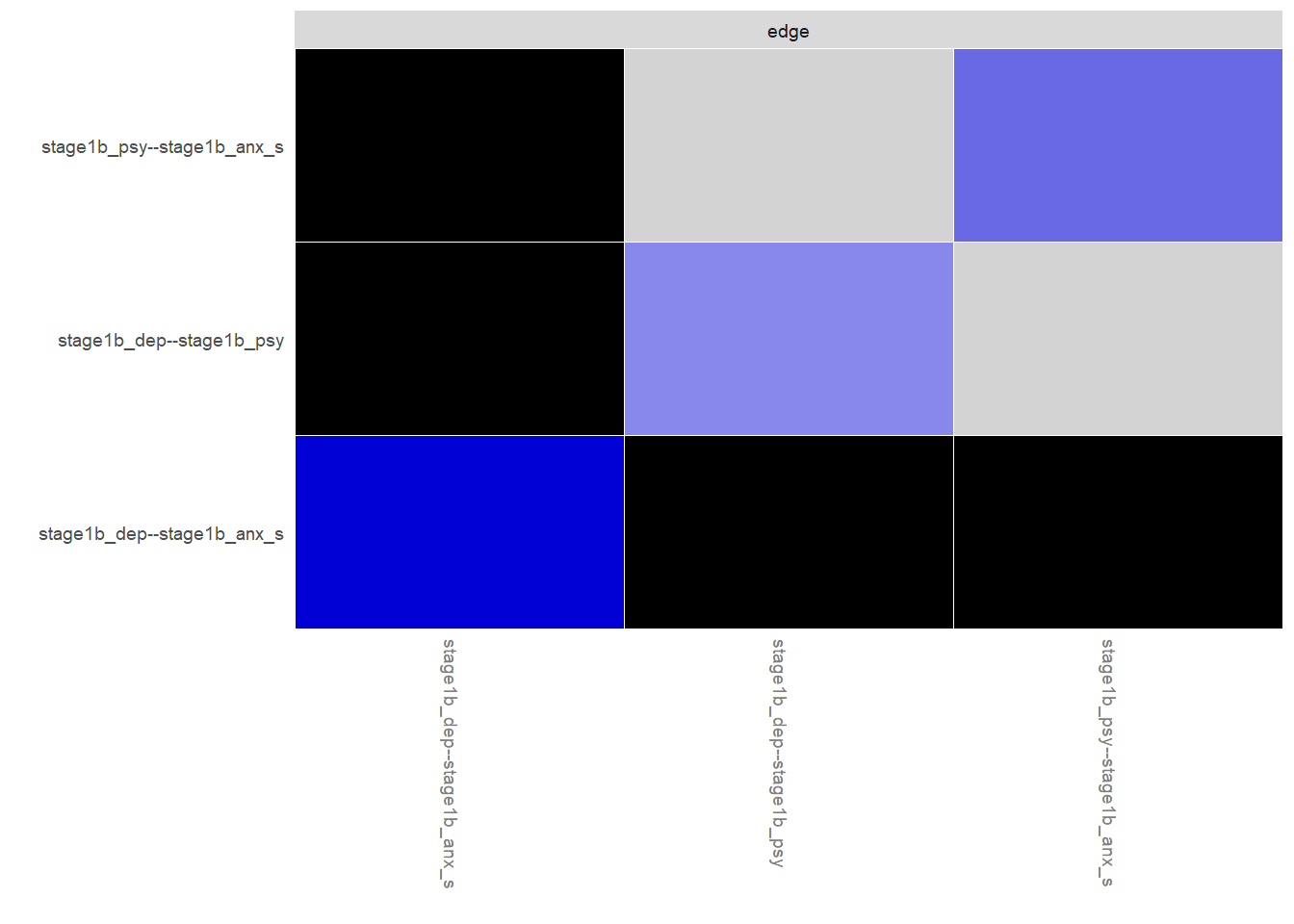 |

Supplementary Figure 3. Bootstrapped difference tests (a = 0.05) between edge-weights that were non-zero in the estimated Ising network of Stage 1b. Gray boxes indicate edges that do not differ significantly from one another and black boxes represent edges that do differ significantly from one another. Saturation of blue boxes on diagonal indicates relative strength of nodes. (A) Using lower-threshold anxiety; (B) Using higher-threshold anxiety.

Notes: stage1b_psy= Stage 1b Psychosis; stage1b_dep= Stage 1b Depression; stage1b_anx= Stage 1b anxiety; stage1b_anx_s= Stage 1b anxiety Higher Threshold.


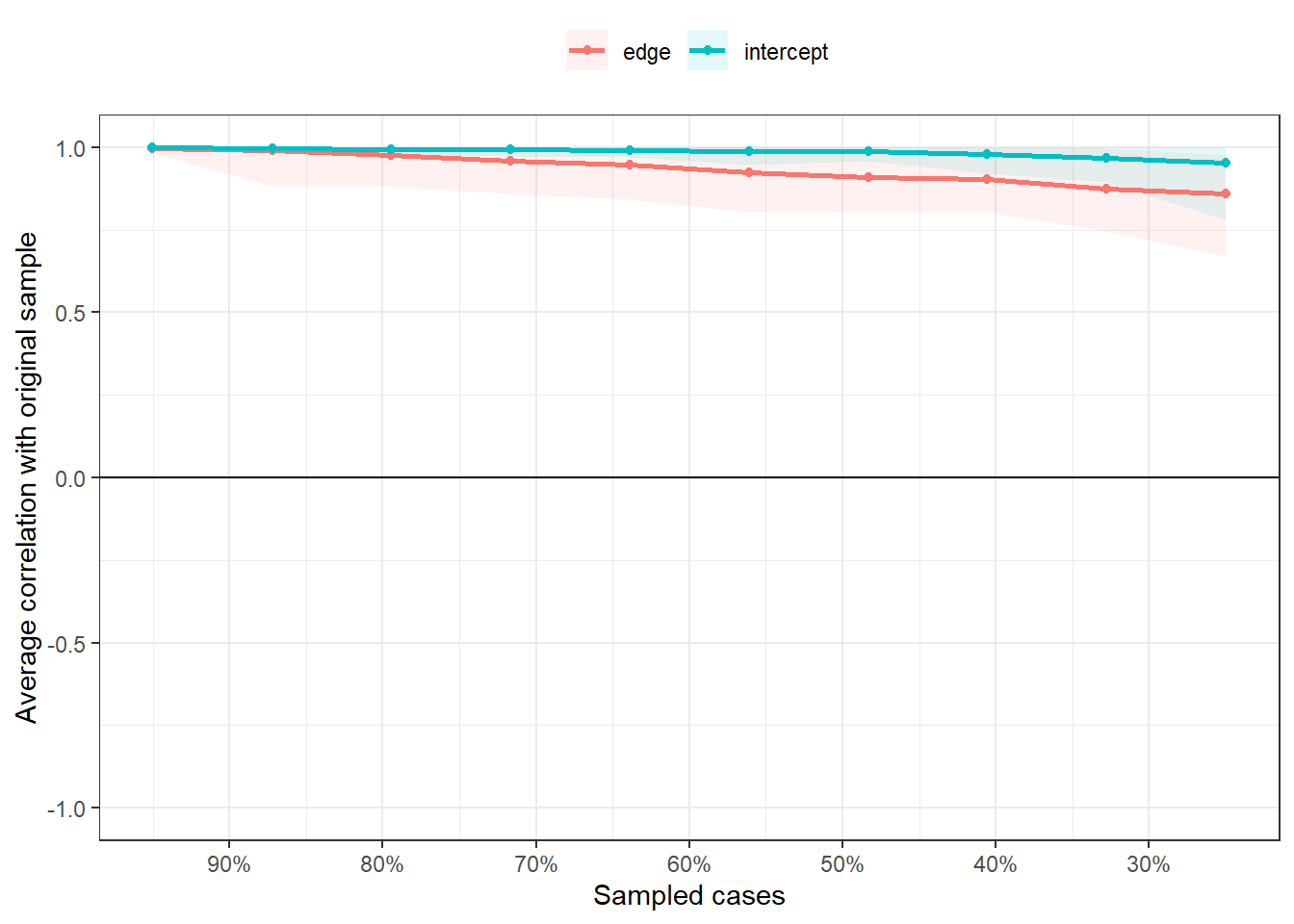


Supplementary Figure 4. Average correlation between original parameters and those found when dropping a successively larger proportion of cases from boot-strapped samples. Lines indicate mean over boot-strapped samples and areas indicate the range from the 2.5th quantile to the 97.5th quantile. Note, parameter stability was almost identical using lower and higher thresholds for anxiety, results using lower-threshold anxiety displayed in this figure.

## Sensitivity Analyses

We conducted all analyses excluding only those with Stage 2 Bipolar type I, rather than those with either Stage 2 Bipolar type I or II. This was to ensure that we did not exclude the possibility of overlap between hypomania and depression due to the definition of Stage 2+ Bipolar II which included those with depression. However, this had no noticeable difference on any results.

**References**

1. Scott J, Leboyer M, Hickie I, Berk M, Kapczinski F, Frank E *et al.* Clinical staging in psychiatry: a cross-cutting model of diagnosis with heuristic and practical value. *Br J Psychiatry* 2013; **202:** 243-245.

2. Shah JL, Scott J, McGorry PD, Cross SPM, Keshavan MS, Nelson B *et al.* Transdiagnostic clinical staging in youth mental health: a first international consensus statement. *World Psychiatry* 2020; **19**(2)**:** 233-242.

3. Iorfino F, Scott EM, Carpenter JS, Cross SP, Hermens DF, Killedar M *et al.* Clinical Stage Transitions in Persons Aged 12 to 25 Years Presenting to Early Intervention Mental Health Services With Anxiety, Mood, and Psychotic Disorders. *JAMA Psychiatry* 2019.

4. Hartmann JA, Nelson B, Spooner R, Paul Amminger G, Chanen A, Davey CG *et al.* Broad clinical high-risk mental state (CHARMS): Methodology of a cohort study validating criteria for pluripotent risk. *Early Interv Psychiatry* 2017.

5. Addington J, Liu L, Goldstein BI, Wang J, Kennedy SH, Bray S *et al.* Clinical staging for youth at-risk for serious mental illness. *Early Interv Psychiatry* 2019; **13**(6)**:** 1416-1423.

6. Sheldrick RC, Benneyan JC, Kiss IG, Briggs-Gowan MJ, Copeland W, Carter AS. Thresholds and accuracy in screening tools for early detection of psychopathology. *J Child Psychol Psychiatry* 2015; **56**(9)**:** 936-948.

7. Cornish RP, John A, Boyd A, Tilling K, Macleod J. Defining adolescent common mental disorders using electronic primary care data: a comparison with outcomes measured using the CIS-R. *BMJ Open* 2016; **6**(12)**:** e013167.

8. Zammit S, Kounali D, Cannon M, David AS, Gunnell D, Heron J *et al.* Psychotic experiences and psychotic disorders at age 18 in relation to psychotic experiences at age 12 in a longitudinal population-based cohort study. *Am J Psychiatry* 2013; **170**(7)**:** 742-750.

9. Ware JE, Jr., Sherbourne CD. The MOS 36-item short-form health survey (SF-36). I. Conceptual framework and item selection. *Med Care* 1992; **30**(6)**:** 473-483.

10. Angst J, Adolfsson R, Benazzi F, Gamma A, Hantouche E, Meyer TD *et al.* The HCL-32: towards a self-assessment tool for hypomanic symptoms in outpatients. *Journal Of Affective Disorders* 2005; **88**(2)**:** 217-233.

11. Lee K, Oh H, Lee EH, Kim JH, Kim JH, Hong KS. Investigation of the clinical utility of the hypomania checklist 32 (HCL-32) for the screening of bipolar disorders in the non-clinical adult population. *BMC Psychiatry* 2016; **16:** 124.

12. Anderson JJ, Hoath S, Zammit S, Meyer TD, Pell JP, Mackay D *et al.* Gestational influenza and risk of hypomania in young adulthood: prospective birth cohort study. *J Affect Disord* 2016; **200:** 182-188.

13. Marwaha S, Winsper C, Bebbington P, Smith D. Cannabis Use and Hypomania in Young People: A Prospective Analysis. *Schizophr Bull* 2018; **44**(6)**:** 1267-1274.

14. Court H, Forty L, Jones L, Gordon-Smith K, Jones I, Craddock N *et al.* Improving the psychometric utility of the hypomania checklist (HCL-32): a Rasch analysis approach. *J Affect Disord* 2014; **152-154:** 448-453.

15. Richards A, Horwood J, Boden J, Kennedy M, Sellers R, Riglin L *et al.* Associations between schizophrenia genetic risk, anxiety disorders and manic/hypomanic episode in a longitudinal population cohort study. *Br J Psychiatry* 2019; **214**(2)**:** 96-102.

16. Lewis G, Pelosi AJ, Araya R, Dunn G. Measuring psychiatric disorder in the community: a standardized assessment for use by lay interviewers. *Psychol Med* 1992; **22**(2)**:** 465-486.

17. World Health Organization. *The ICD‐10 classification of mental and behavioural disorders: clinical descriptions and diagnostic guidelines.* World Health Organisation: Geneva, 1992.

18. Turner N, Joinson C, Peters TJ, Wiles N, Lewis G. Validity of the Short Mood and Feelings Questionnaire in late adolescence. *Psychol Assess* 2014; **26**(3)**:** 752-762.

19. Hogberg G, Hallstrom T. Mood Regulation Focused CBT Based on Memory Reconsolidation, Reduced Suicidal Ideation and Depression in Youth in a Randomised Controlled Study. *Int J Environ Res Public Health* 2018; **15**(5).

20. Skapinakis P, Bellos S, Koupidis S, Grammatikopoulos I, Theodorakis PN, Mavreas V. Prevalence and sociodemographic associations of common mental disorders in a nationally representative sample of the general population of Greece. *BMC Psychiatry* 2013; **13:** 163.

21. Spitzer RL, Kroenke K, Williams JB, Lowe B. A brief measure for assessing generalized anxiety disorder: the GAD-7. *Arch Intern Med* 2006; **166**(10)**:** 1092-1097.

22. Merikangas KR, He JP, Burstein M, Swanson SA, Avenevoli S, Cui L *et al.* Lifetime prevalence of mental disorders in U.S. adolescents: results from the National Comorbidity Survey Replication--Adolescent Supplement (NCS-A). *J Am Acad Child Adolesc Psychiatry* 2010; **49**(10)**:** 980-989.

23. Yung AR, Yuen HP, McGorry PD, Phillips LJ, Kelly D, Dell'Olio M *et al.* Mapping the onset of psychosis: the Comprehensive Assessment of At-Risk Mental States. *The Australian and New Zealand journal of psychiatry* 2005; **39**(11-12)**:** 964-971.

24. Goodday SM, Preisig M, Gholamrezaee M, Grof P, Angst J, Duffy A. The association between self-reported and clinically determined hypomanic symptoms and the onset of major mood disorders. *BJPsych Open* 2017; **3**(2)**:** 71-77.

25. Office for National Statistics. *Standard Occupational Classification 2000*, vol. 1, Structure and descriptors of unit groups. The Stationery Office: London, 2000.

26. Houtepen LC, Heron J, Suderman MJ, Tilling K, Howe LD. Adverse childhood experiences in the children of the Avon Longitudinal Study of Parents and Children (ALSPAC). *Wellcome Open Res* 2018; **3:** 106.

27. Zammit S, Odd D, Horwood J, Thompson A, Thomas K, Menezes P *et al.* Investigating whether adverse prenatal and perinatal events are associated with non-clinical psychotic symptoms at age 12 years in the ALSPAC birth cohort. *Psychol Med* 2009; **39**(9)**:** 1457-1467.

28. Bowen E, Heron J, Waylen A, Wolke D, Team AS. Domestic violence risk during and after pregnancy: findings from a British longitudinal study. *BJOG* 2005; **112**(8)**:** 1083-1089.

29. Goodman R. The Strengths and Difficulties Questionnaire: a research note. *J Child Psychol Psychiatry* 1997; **38**(5)**:** 581-586.

30. Young C, Craig JC, Clapham K, Banks S, Williamson A. The prevalence and protective factors for resilience in adolescent Aboriginal Australians living in urban areas: a cross-sectional study. *Aust N Z J Public Health* 2019; **43**(1)**:** 8-14.

31. Savage-McGlynn E, Redshaw M, Heron J, Stein A, Quigley MA, Evans J *et al.* Mechanisms of Resilience in Children of Mothers Who Self-Report with Depressive Symptoms in the First Postnatal Year. *PLoS One* 2015; **10**(11)**:** e0142898.

32. Saunders JB, Aasland OG, Babor TF, de la Fuente JR, Grant M. Development of the Alcohol Use Disorders Identification Test (AUDIT): WHO Collaborative Project on Early Detection of Persons with Harmful Alcohol Consumption--II. *Addiction* 1993; **88**(6)**:** 791-804.

33. Allen JP, Litten RZ, Fertig JB, Babor T. A review of research on the Alcohol Use Disorders Identification Test (AUDIT). *Alcohol Clin Exp Res* 1997; **21**(4)**:** 613-619.

34. Legleye S, Guignard R, Richard JB, Ludwig K, Pabst A, Beck F. Properties of the Cannabis Abuse Screening Test (CAST) in the general population. *Int J Methods Psychiatr Res* 2015; **24**(2)**:** 170-183.

35. Slopen N, Williams DR, Fitzmaurice GM, Gilman SE. Sex, stressful life events, and adult onset depression and alcohol dependence: Are men and women equally vulnerable? *Social Science and Medicine* 2011; **73**(4)**:** 615-622.

36. Hammen C. Stress and depression. vol. 12005, pp 293-319.

37. Tessner KD, Mittal V, Walker EF. Longitudinal study of stressful life events and daily stressors among adolescents at high risk for psychotic disorders. *Schizophr Bull* 2011; **37**(2)**:** 432-441.

38. Crane C, Heron J, Gunnell D, Lewis G, Evans J, Williams JM. Childhood traumatic events and adolescent overgeneral autobiographical memory: findings in a U.K. cohort. *J Behav Ther Exp Psychiatry* 2014; **45**(3)**:** 330-338.

39. Van Borkulo CD, Borsboom D, Epskamp S, Blanken TF, Boschloo L, Schoevers RA *et al.* A new method for constructing networks from binary data. *Scientific reports* 2014; **4**(1)**:** 1-10.
